# Supertransport of excitons in atomically thin organic semiconductors at the 2D quantum limit

**DOI:** 10.1038/s41377-020-00347-y

**Published:** 2020-07-06

**Authors:** Ankur Sharma, Linglong Zhang, Jonathan O. Tollerud, Miheng Dong, Yi Zhu, Robert Halbich, Tobias Vogl, Kun Liang, Hieu T. Nguyen, Fan Wang, Shilpa Sanwlani, Stuart K. Earl, Daniel Macdonald, Ping Koy Lam, Jeffrey A. Davis, Yuerui Lu

**Affiliations:** 1 Research School of Electrical, Energy and Materials Engineering, College of Engineering and Computer Science, The Australian National University, Canberra, ACT 2601 Australia; 2 Optical Sciences Centre, Swinburne University of Technology, Hawthorn, VIC 3122 Australia; 3 ARC Centre of Excellence for Future Low-Energy Electronics Technology, Australia; 4 Centre for Quantum Computation and Communication Technology, Department of Quantum Science, Research School of Physics and Engineering, The Australian National University, Acton, ACT 2601 Australia; 5 School of Mechatronical Engineering, Beijing Institute of Technology, Beijing, 100081 China; 6 Institute for Biomedical Materials and Devices (IBMD), Faculty of Science, University of Technology Sydney, Sydney, NSW 2007 Australia

**Keywords:** Ultrafast photonics, Organic LEDs

## Abstract

Long-range and fast transport of coherent excitons is important for the development of high-speed excitonic circuits and quantum computing applications. However, most of these coherent excitons have only been observed in some low-dimensional semiconductors when coupled with cavities, as there are large inhomogeneous broadening and dephasing effects on the transport of excitons in their native states in materials. Here, by confining coherent excitons at the 2D quantum limit, we first observed molecular aggregation-enabled ‘supertransport’ of excitons in atomically thin two-dimensional (2D) organic semiconductors between coherent states, with a measured high effective exciton diffusion coefficient of ~346.9 cm^2^/s at room temperature. This value is one to several orders of magnitude higher than the values reported for other organic molecular aggregates and low-dimensional inorganic materials. Without coupling to any optical cavities, the monolayer pentacene sample, a very clean 2D quantum system (~1.2 nm thick) with high crystallinity (J-type aggregation) and minimal interfacial states, showed superradiant emission from Frenkel excitons, which was experimentally confirmed by the temperature-dependent photoluminescence (PL) emission, highly enhanced radiative decay rate, significantly narrowed PL peak width and strongly directional in-plane emission. The coherence in monolayer pentacene samples was observed to be delocalised over ~135 molecules, which is significantly larger than the values (a few molecules) observed for other organic thin films. In addition, the supertransport of excitons in monolayer pentacene samples showed highly anisotropic behaviour. Our results pave the way for the development of future high-speed excitonic circuits, fast OLEDs, and other optoelectronic devices.

## Excitonics: Supertransport in 2D organic semiconductor

Scientists have detected excited quasi-particles, called excitons, moving far and fast in a two-dimensional organic semiconductor, paving the way for applications in quantum computing and fast light emitting diodes. Yuerui Lu of the Australian National University and colleagues conducted tests in a 2D sample of pentacene, an organic semiconducting compound made of benzene rings fused into a chain. Light particles are absorbed by pentacene, generating excitons that can be used to develop tiny, very fast, energy-efficient optoelectronic devices. The team found that excitons move through the crystal depending on the temperature and pentacene’s molecular arrangements and order of crystallinity. The long-range and fast migration of excitons in a monolayer of pentacene was at least an order of magnitude higher than in other materials. The excitons in the pentacene monolayer also generated sharp, strong ‘superradiant’ light emissions.

## Introduction

Recently, there has been increasing interest in harnessing the long-range and fast transport of coherent excitons (electron-hole pair quasi-particles) in solid-state inorganic semiconductors and molecular systems with confined geometries to enhance light-matter interactions^1–3^. Coherence is critical to engineering the quantum electrodynamics (QED)^4^ in low-dimensional systems, such as quantum wells^5^, two-dimensional (2D) materials^6^, and quantum dots^7^, leading to various applications in realising quantum memories^8^, single-photon sources^9^, laser cooling^10^, narrow linewidth lasers^11^, high efficiency solar cells^12^, and stable polaritons to achieve Bose Einstein condensates^5^. Long-range and fast migration of coherent excitons also has tremendous applications in schemes of high-speed excitonic circuits, quantum computing^13–15^, and high quantum yield light-emitting diodes^16^.

Spontaneous coherent emission from a system of several non-interacting dipole active atoms (excitons) was defined as superradiance (SR) by Dicke^17^. In this phenomenon, interactions between transition dipoles of individual molecules allow coherent delocalisation across multiple sites. This leads to a net enhancement in the optical transition dipole moment (TDM) and a sharp enhancement of the excitonic radiative decay rate of an ensemble of *N*_*c*_ independent emitters compared to the radiative decay rate of a single emitter^2,7^. The same principle of coherent delocalised superradiant emission gives rise to an analogous phenomenon called cooperative energy transfer or supertransfer (ST)^18^. The resulting enhanced oscillator strength from delocalisation over large molecular assemblies can lead to large-scale exciton transport. In an ST process, the molecular assemblies (consisting of *N*_*c*_ molecules) with comparable net TDMs can play the role of acceptors similar to individual molecules in the Förster resonance energy transfer (FRET) mechanism^19^. Thus, the excitation (exciton) can be transferred over much longer distances in a delocalised molecular assembly before annihilation, resulting in large effective exciton diffusion coefficients.

Recently, coherent exciton transport with highly enhanced effective exciton diffusion coefficients (3–70 cm^2^/s)^20–22^ was observed in quasi-one-dimensional (quasi-1D) molecular assemblies (cylindrical bundles^20^, nanotubes^19^ and wires^21,22^), and these coefficients were significantly higher than the migration speeds of incoherent excitons reported in other materials, including conventional organic thin films and their heterostructures (0.001–3 cm^2^/s)^23,24^, III–V semiconductor quantum wells (0.1–10 cm^2^/s)^25^, atomically thin transition metal dichalcogenides (TMDs) and phosphorene (0.01–14.5 cm^2^/s)^26^, etc. (Table S1 in the Supplementary Information). However, the migration speeds of the coherent excitons in these quasi-1D systems^20–22^ were still hindered by the low overall exciton oscillation strengths (low coherence length with small *N*_c_ values), which were mainly limited by the low quantum confinement of excitons^27^ (diameter size >12 nm) and potential disorder and interfacial sites in the systems^28^. Additionally, these quasi-1D systems had very small cross-section areas for light-matter interactions, limiting their applications in future excitonic devices^3,29–31^. As predicted by theory, the exciton oscillation strength, a key parameter for coherent excitons, is very sensitive to the quantum confinement of the system^27^. Therefore, confining coherent excitons at the 2D quantum limit is a very promising way to realise long-range and fast transport of excitons; moreover, the 2D structure can also provide a large cross-section area for light-matter interactions, enabling tremendous applications in future excitonic devices.

In this work, in atomically thin organic molecular crystals without any optical cavities, we observe long-range and fast migration of Frenkel (FR) excitons between coherent states at the 2D quantum limit. A very high effective exciton diffusion coefficient of ~346.9 cm^2^/s at room temperature is reported, which is one order of magnitude higher than the values previously reported (Table S1 in the Supplementary Information) for other materials^20,22,32^. The sharp, strong emission arising from J-type (monolayer, shortened as 1L) aggregation in pentacene in contrast to the emission arising from H-type (wetting layer, shortened as WL) aggregation is confirmed to be superradiant emission from FR excitons. The coherence in 1L pentacene samples is determined to be delocalised over ~135 molecules, which is more than one order of magnitude larger than the values (a few molecules) observed in other organic thin films^33^. Our simulation results from quantum calculations attribute this to the constructive dipole coupling in the J-aggregates in 1L pentacene that forms an enhanced net optical dipole moment, which supports our experimental observations. The supertransport of excitons observed on the macroscopic scale will enable realisation of strongly enhanced light-matter interactions at the quantum limit using such 2D organic materials, which has key applications in developing high-speed quantum computing devices, fast response time OLEDs, excitonic transistors and other optoelectronic devices^8,15,31^.

## Results

### Sample growth and characterization

**Fig. 1:**
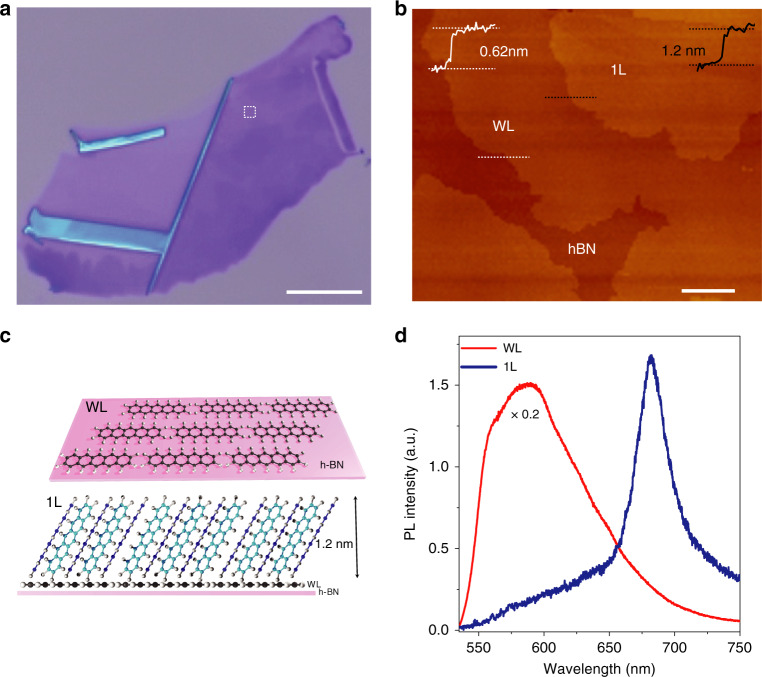
Characterization of atomically thin 2D layered pentacene samples. **a** Optical microscope image of the sample used for measurements. The scale is 80 μm. **b** Zoomed-in atomic force microscopy (AFM) image of the dashed square region in **a**, showing the actual measured thicknesses of WL and 1L pentacene. The scale is 2 μm. **c** Schematic diagram showing the orientation and alignment of pentacene molecules in WL and 1L over h-BN and a SiO_2_/Si substrate. **d** Measured PL spectra from WL and 1L samples at room temperature

Single-crystal pentacene (Fig. 1) was epitaxially grown layer by layer on a hexagonal boron nitride (h-BN) surface with atomic smoothness and well-defined crystal facets. The first pentacene layer on h-BN was named the wetting layer (WL; ~0.6 nm thick), and the next layer of pentacene grown on WL was designated the monolayer (1L; ~1.2 nm thick)^34^ (Fig. 1a–c). The layer-dependent arrangement of pentacene molecules over h-BN and their electrical transport properties were described in a recent report^34^. Raman spectroscopy was used to confirm the presence of pentacene on h-BN (Fig. S1). PL spectroscopy with a continuous-wave 532 nm excitation laser was used to characterise the excitonic emissions from the atomically thin pentacene layers. The WL region showed much stronger PL emissions than the 1L region at room temperature (Figs. 1d and S2 and associated text). Compared with inorganic TMD 2D semiconductors, WL showed a much broader PL spectrum at room temperature, which is associated with the various band energy levels formed in pentacene due to vibronic couplings between FR and charge-transfer (CT) excitons (Supplementary Information note 1 and Fig. S3)^35,36^. On the other hand, the 1L region showed a single peak centred at ~680 nm that corresponds predominantly to FR excitonic emission, which will be explained later. This region is a heterostructure consisting of 1L on top of WL. However, in the PL spectrum from the 1L region, we did not see the higher-energy emission (~ 550–650 nm) observed in the WL sample (Fig. 1d) because the photoexcited charges in the WL underneath could be quickly transferred to 1L before radiative emission occurred (Fig. S2). Therefore, the PL spectrum measured from the 1L region comes from the 1L pentacene sample and reflects its intrinsic nature.

### Superradiance and excitonic characteristics

**Fig. 2:**
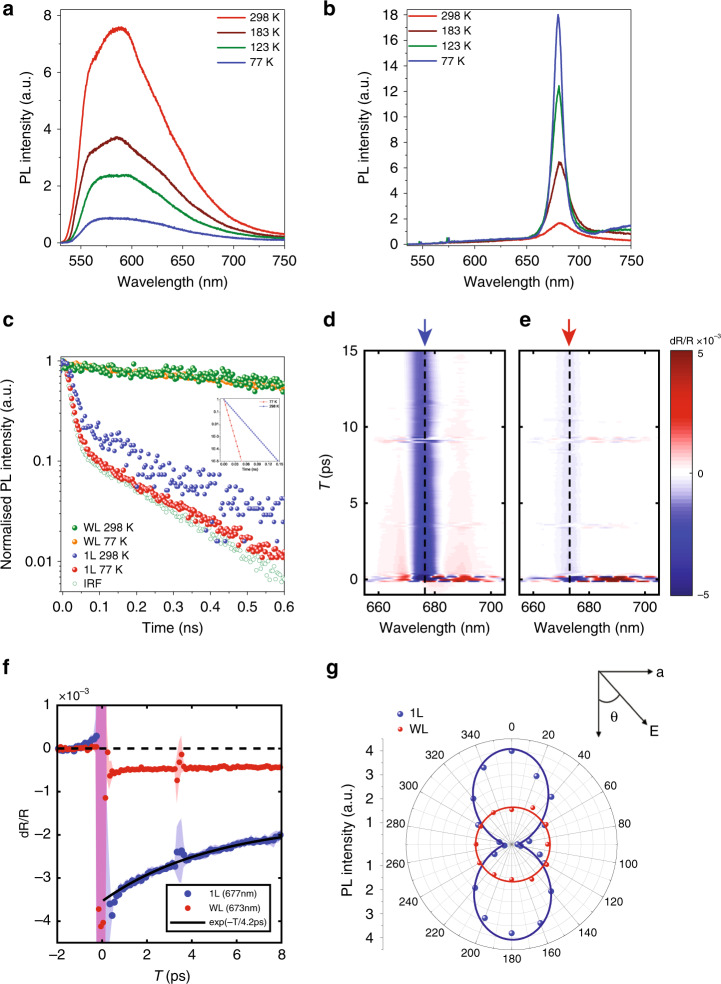
Temperature-dependent optical measurements. **a**, **b** PL spectra measured at various temperatures for WL (**a**) and 1L (**b**). For 1L, a sharp peak at ~680 nm (designated as FR excitonic emission) rises sharply as the temperature decreases. **c** Time-resolved PL emission (normalised) from WL and 1L pentacene samples at 77 and 298 K. The orange/green balls represent the measured decay curve from WL, which arises from CT exciton emission. An effective long lifetime of 2.64 ns (2.89 ns) was extracted from the orange (green) decay curve at 77 K (298 K) by fitting with deconvolution of the instrument response function (IRF) (green dots). The red/blue balls represent the decay curve from 1L, which arises from FR exciton emission. The red (blue) decay curve was fitted (solid line) with deconvolution of the IRF, giving an effective short lifetime of 4.1 ps (12.7 ps) for 1L at 77 K (298 K). The inset shows the deconvoluted curves obtained from 1L. **d**, **e** Time- and spectrally resolved transient differential reflectance of 1L (**c**) and WL (**d**) recorded using ~200 fs degenerate pump and probe with spectra centred at 680 nm. **f** Slices of the TR for 1L (blue) and WL (red) at 677 nm and 673 nm, respectively. A lifetime of 4.2 ps was extracted from the 1L decay, while the WL response was effectively constant across the range. The feature at 3.5 ps is due to interference between the pump pulse and a weak reflection of the probe pulse. **g** Measured polar plot of PL intensity as a function of emission polarisation angle *θ* from 1L (blue) and WL (red) pentacene samples at 77 K, revealing the anisotropic (isotropic) excitonic nature of emission from 1L (WL) pentacene. In the experiment, the excitation polarisation angle was fixed, and the polarisation angle of the emission *θ* was determined by an angle-variable polarizer located in front of the detector. The solid lines are curves fitted using a cos^2^*θ* function

To explore the nature of excitonic transitions, we conducted temperature-dependent PL measurements on the WL and 1L pentacene samples down to 77 K (Fig. 2). With decreasing temperature, the PL intensity from WL dropped significantly, and the full-width at half-maximum (FWHM) broadened (Fig. 2a); in contrast, 1L pentacene showed a sharp rise in the PL intensity and a significantly reduced FWHM with decreasing temperature (Figs. 2b and S4). The opposing temperature dependences were caused by the completely different molecular packings in WL and 1L pentacene, which can be fully understood based on the theory of H- and J-type molecular aggregation and their effects on optical emission^35,37^ (Supplementary Information note 2; Fig. S3). Theoretical values of the coupling coefficients (*J*) and net TDM in each layer were extracted based on the molecular aggregation pattern and coupling (Fig. S3a). WL exhibited a parallel packing of TDMs, and 1L exhibited a head-to-tail packing of TDMs because of the herringbone packing of pentacene molecules in the unit cell (Figs. 1c and S3)^38^. The *J* values for 1L and WL pentacene were negative and positive, respectively, suggesting J- and H-type aggregation for 1L and WL, respectively^38^. The total TDM and oscillator strength for the lowest excited states (*S*_1_) in WL were both zero, leading to non-luminescent *S*_1_ states at a temperature of absolute zero. In contrast, the total TDM for *S*_*1*_ in 1L had a large value (with the direction along the *b* axis of the unit cell), and its corresponding oscillator strength was high (Fig. S3a). This resulted in luminescent *S*_*1*_ states at absolute zero. The large magnitude of the net TDM corresponds to phase locking and constructive coupling of TDMs in 1L pentacene cooperatively, resulting in a sharp rise in the PL intensity and a reduction in the FWHM, which are characteristics of superradiant emission^33,35,39^.

The phenomenon of superradiance is the combined process of coherent emission from a cluster of *N*_c_ molecular aggregates/chromophores whose oscillator strength is enhanced compared to that of a single emitter by a factor of *N*_c_ owing to the large TDM associated with the molecular assembly^33,37^. To determine which coherent size gives the best agreement with the experimental temperature dependence of the PL intensity, we used the Huang-Rhys factor and the spectral strengths of the 0-0 and 0–1 transitions in the 1L PL spectrum to accurately determine the coherence number and hence the effect of vibrations and phononic interactions on the coherence length^36^. The *N*_c_ value can also be estimated by the sharp reduction in the FWHM and spectral strengths of the PL emission as the temperature decreases^39^. Based on this technique and our measurements in Figs. 2c, S4 and S5, we estimated this value to be over 135 molecules. (See Supplementary Information note 3.) The extracted *N*_c_ value for our 1L pentacene sample was more than one order of magnitude larger than that observed for tetracene thin film (*N*_c_ ~ a few molecules)^33^. The *N*_c_ value signifies the extent of FR exciton delocalisation, which was over 135 consecutive dipoles in 1L pentacene. The enhanced oscillator strength was distributed over these consecutive dipoles, resulting in a large net TDM, as shown in Fig. S3.

Excitonic superradiance also leads to shortening of the excitonic radiative decay lifetime for the emission^2,4,7,11,33,39^ by a factor of *N*_c_. To confirm this coherent superradiance from 1L, we measured the temperature-dependent lifetimes of two excitonic emissions from WL and 1L pentacene samples using time-resolved photoluminescence (TRPL) measurements with a resolution of 2.1 ps after deconvolution (Fig. S6). The lifetime observed for the 1L pentacene sample significantly dropped from ~12.7 ± 0.8 ps at room temperature to ~4.1 ± 0.7 ps at 77 K (Fig. 2c). To further confirm the measurement accuracy of the lifetime, we performed time- and spectrally resolved differential transient reflectance (TR) measurements on WL and 1L pentacene samples at 77 K using a pump-probe technique with a time resolution of 200 fs. The 1L pentacene sample showed a clear dip in the differential TR mapping centred at 677 nm; in contrast, the dip was not clear for the WL sample (Fig. 2d, e). The relaxation of this feature in the 1L sample followed a fast decay (lifetime of ~4.2 ± 0.53 ps; Fig. 2f). Fitting of the decay curve was performed using a single exponential decay and a 95% confidence interval, giving an estimated lifetime range of 3.6–4.7 ps (Fig. S7). Further details about the measurements and the fitting are explained in Supplementary Information Fig. S7 and the associated text. The consistency between the two complementary techniques confirms the accuracy of our lifetime values measured with our TRPL system. The significant reduction in the emission lifetime with decreasing temperature is consistent with the observed sharp rise in the PL intensity in the 1L pentacene sample (Fig. 2b), confirming the fast and radiative nature of the decay, as expected for superradiance.

Furthermore, the radiative lifetime of singlet FR excitonic emissions in bulk pentacene thin films (which are non-coherent monomer states) was experimentally established to be ~1200 ps^40^. We also confirmed the lifetime in our bulk thin film pentacene grown on the same h-BN substrate, which was measured to be 1241.1 ps at room temperature (Fig. S8 and associated text). The significantly reduced lifetime (~12.7 ps) in our 1L pentacene sample compared to the value (~1200 ps) for the non-coherent states in thin film pentacene, coupled with the consistent temperature-dependent PL emission and lifetime, further substantiates superradiant emission from 1L pentacene. The value of *N*_c_, as extracted from this sharp reduction in lifetime, was ~100 at room temperature, which is again in close agreement with the aforementioned extracted values based on the highly enhanced PL intensity and reduced FWHM with decreasing temperature. In contrast, the lifetimes for the WL pentacene sample were measured to be 2.89 ns using TRPL at room temperature and 2.64 ns at 77 K (Fig. 2c), much larger than the values from the 1L pentacene samples, confirming the non-coherent nature of the CT excitons in the WL samples.

We further performed PL measurements with angle-resolved excitation and emission polarisation (Figs. 2g and S9) to confirm the anisotropic nature of the superradiant FR excitonic emissions from 1L. The PL intensity from 1L strongly depended on the emission polarisation angle *θ* and showed a period of 180°; in contrast, the emission from WL was isotropic (Fig. 2g). The theoretically predicted strongly directional total TDM in 1L pentacene (Fig. S3a) is expected to lead to an in-plane anisotropic emission of the FR excitons. Henceforth, the zero-degree reference in the polar plots (Fig. 2f) and subsequent figures can be identified as the *b* axis of the unit cell in 1L pentacene (Figs. 1c and S3a). The directional emission in the 1L pentacene sample was independent of the excitation polarisation angle of the incident laser (Fig. S9). A similar anisotropy for the PL emission from 1L pentacene was observed for 1L samples at room temperature (Fig. S10). Normally, the dichroic ratio (*DR*) is a term used to characterise the in-plane anisotropy of optical transitions^41^. Here, it is defined as DR = I_∥_/I_⊥_, where I_∥_ and I_⊥_ are the PL peak intensities measured at polarisation angles of 0° and 90°, respectively. The measured DR values of PL emission from 1L for emission (Fig. 2g, blue curve) polarisation at 77 K were high, up to 10.3, which indicates the strongly anisotropic nature of the PL emission from 1L. At room temperature, the DR value observed for 1L samples was close to 5.9. In sharp contrast, the DR values measured from the PL emission in WL pentacene were isotropic both at room temperature and 77 K (Fig. 2g, red curve), which suggests that these peaks are dominated by the contribution of CT excitons^42^. Supplementary Information note 4 presents a further detailed discussion on the different natures of the excitonic states in WL and 1L.

### Long-range and superfast exciton transport

**Fig. 3:**
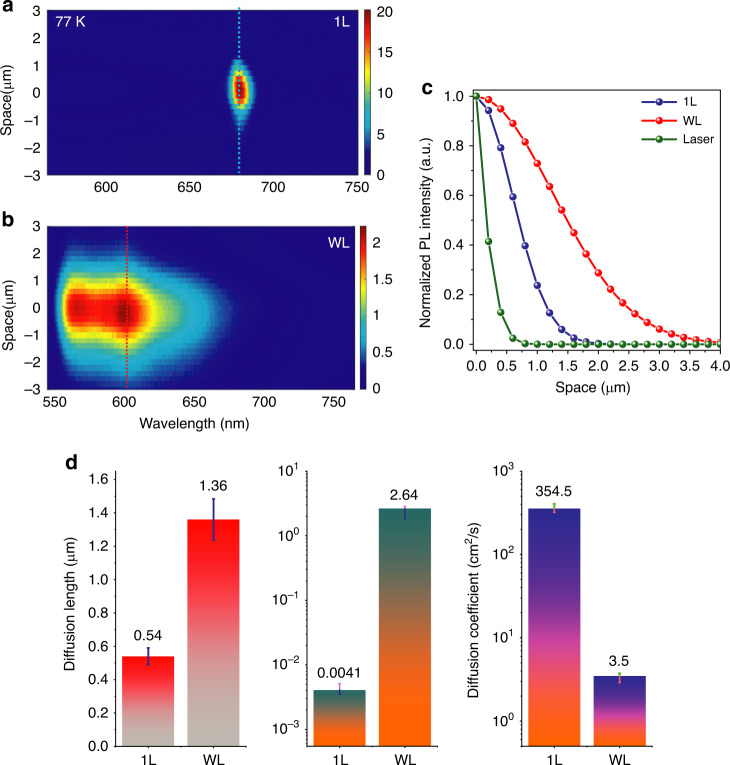
Exciton transport mapping using near-field imaging. **a**, **b** Measured contour plots of PL intensity as a function of emission wavelength and space of exciton diffusion for 1L (**a**) and WL (**b**) pentacene samples at 77 K. The middle of the laser excitation spot is at *x* = 0. **c** Spatial profiles plotted along the dotted lines in **a**, **b** for different types of excitonic peaks, showing the diffusion lengths of excitons in WL and 1L. **d** Comparison plots of diffusion lengths (left; from Fig. 3a–c), lifetime (middle; from Fig. 2e) and extracted diffusion coefficients measured from WL and 1L samples at 77K. The error bars represent the experimental variation observed in measurements of multiple samples

**Fig. 4:**
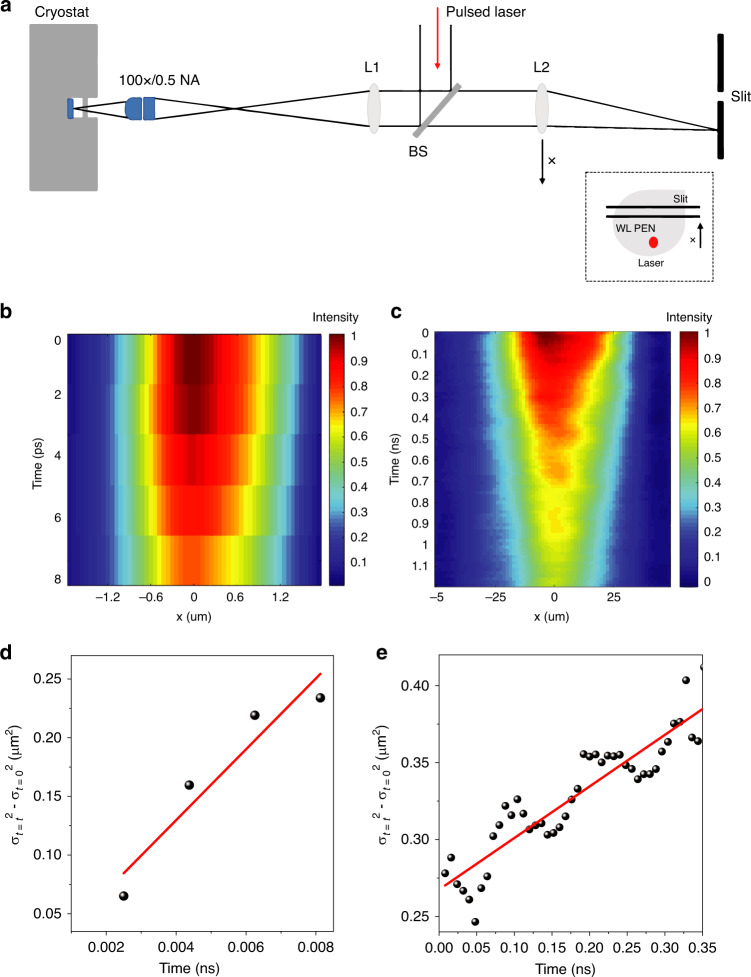
Spatial-temporal mapping of 1L and WL PEN. **a** Schematic showing the experimental setup with a movable lens used for mapping. A pulsed 522-nm laser is focused onto 1L/WL placed on the copper cold finger in a cryostat using a beam splitter and a ×100/0.5-NA-long working distance objective. Photoluminescence from the sample is imaged onto the entrance slit of the spectrometer using lenses L1 and L2. Lens L2 is translated to position the WL emission onto the entrance slit of the spectrometer. **b**, **c** Spatial-temporal mapping for 1L (WL), showing the diffusion in time and space. **d**, **e** Time evolution of the mean square displacement of excitons in space for 1L (WL). The diffusion coefficients obtained for 1L and WL were 306.8 ± 14.1 and 3.3 ± 1.1 cm^2^/s, respectively, confirming the diffusion length observed from Fig. 3b

The same principle of coherent delocalised superradiant emission gives rise to an analogous phenomenon called cooperative energy transfer or supertransfer^18^. The enhanced oscillator strength resulting from delocalisation over large molecular assemblies can lead to large-scale exciton transport. The near-field direct imaging technique with a charge-coupled device (CCD) camera^21,22,43^ was used to detect the diffusion length of bright excitons from WL and 1L pentacene samples (Fig. 3). Figure 3a, b shows the measured contour plots of the PL intensity as a function of the emission wavelength and space of exciton diffusion for the 1L (WL) pentacene sample at 77 K. The spatial distribution of the PL intensity can be extracted at specific energies (shown by dotted lines in Fig. 3a, b). Figure 3c shows the extent of spatial diffusion in a steady 1D diffusion model. A Gaussian fitting routine^21,43^ was used to quantitatively compare the reflected excitation beam to the emission profile emerging from pentacene. Both the emission and laser profiles were fitted using a bivariate normal distribution, and their FWHMs were extracted. The spatial extent of exciton diffusion (diffusion length *L*_*D*_) was extracted by taking the difference between the standard deviations of the emission image and the passive laser spot after fitting the raw data. A Gaussian fitting model (see methods) was used because the excitation from the laser profile was predominantly Gaussian and the diffusion model was not pair mediated or defect assisted in our high crystalline sample^44,45^. The exciton diffusion lengths (*L*_*D*_) in the 1L and WL pentacene samples were measured to be 0.55 ± 0.06 μm and 1.36 ± 0.16 μm, respectively (Fig. 3d). All the measurements were taken at a low excitation power of ~6 μW. We also performed measurements with different powers ranging from 6 to 215 μW, and similar diffusion lengths and lifetimes were observed, which shows the minimal and ignorable effect of exciton-exciton annihilation in our measurements (Fig. S11)^19,46^. Using the measured excitonic emission lifetimes (*τ*), the effective exciton diffusion coefficient (*D*_eff_) could be extracted using the equation^22,46^$L_{D} = \sqrt{2D_{{\mathrm{eff}}}\tau}$. Using the lifetimes (Fig. 2e), the extracted diffusion coefficients for the 1L and WL pentacene samples at 77 K were 354.5 ± 50.1 cm^2^/s and 3.5 ± 0.2 cm^2^/s, respectively (Fig. 3d). The *D*_*eff*_ value obtained from 1L is almost one order of magnitude higher than the values reported for other organic/inorganic systems with coherent and delocalised excitonic emissions (3–70 cm^2^/sec, Table 1 in Supplementary Information)^20–22^. At the same time, this value is ~3 orders of magnitude higher than the diffusion constant reported for non-coherent singlet excitons in bulk thin film pentacene by Marciniak et al.^40^. The exciton diffusion coefficients in WL and 1L at 77 K were further corroborated by another alternative method, spatial-temporal mapping^47^, which is a very robust method for accurately determining the exciton diffusion coefficient in similar materials^47^. Figure 4a shows the experimental setup used for spatial-temporal mapping, and the obtained data plots stitched together are shown in Fig. 4b, c. (See Methods section) The diffusion coefficient extracted from the spatial-temporal mapping was obtained by extracting the Gaussian dispersion of the emission profile over time and was consistent with our reported values obtained from the time-resolved PL and diffusion mapping in space technique, highlighted in Fig. 3. The diffusion coefficients for 1L and WL were 306.8 ± 14.1 cm^2^/s and 3.3 ± 1.1 cm^2^/s, respectively, as obtained from the slope in Fig. 4d, e. It is important to remark here that the initial diffusion value for WL was non-zero due to cooling of hot CT excitons in the <4 ps regime^48^. As discussed earlier in this report, WL is predominantly CT exciton dominated. For CT excitons in the initial few hundred femtoseconds to few ps regime, the CT excitons undergo cooling or lose excess energy due to the creation of many intermediate energy states between S_1_ (natural excited exciton energy state) and CT states, and they are all electronic states that excitons can quickly jump to in a few ps^24,48^. Furthermore, due to excitons residing on two different molecules, the extent of movement is also enhanced. All of this occurs in a few hundred femtoseconds to a few ps; hence, the non-zero value in WL can be attributed to hot CT exciton states cooling rapidly to intermediate energy levels created due to H-type aggregation and donor/acceptor-mediated hopping of excitons. Once the initial cooling occurs, the excitons then stabilise and diffuse normally. Since this non-zero transport occurred within the few ps regime, it did not affect the accuracy of our measurement of the exciton diffusion coefficient in WL, where the diffusion stretched to the few hundred ps-ns regime. In 1L samples, which are predominantly FR exciton dominated, we did not observe such non-zero transport at *t* = 0, as the hot CT excitons were practically non-existent there. The results further confirm the effectiveness of our technique in measuring precise exciton diffusion with a high resolution and the limited role of triplet fission in our samples.

We attribute the high value of the exciton diffusion coefficient in 1L to the supertransfer phenomenon. The reason we were able to achieve these large effective exciton diffusion coefficients is threefold. First, in our 1L pentacene samples, the strong intermolecular coupling (J-type aggregation) results in a strong net exciton oscillator strength and thus delocalised and superradiant excitonic emissions. Within each delocalised segment, the excitation energy propagates ballistically^18^, in contrast to the diffusive-hopping-described FRET. Delocalised excitons in principle accelerate energy transfer compared to incoherent molecular hopping transfer because delocalisation can define an effective hopping length that is much larger than the nearest intermolecular spacings (Supplementary Information note 5)^49–51^. Second, because of the high-quality epitaxial 2D growth, our pentacene samples have highly ordered crystallinity and minimised defects/disorder, resulting in long delocalisation lengths and large coherence lengths (*N*_c_ ~ 135, as shown in Fig. S5, extracted from temperature-dependent PL line strength measurements), which significantly benefit the transport speed of coherent excitons. The exciton delocalisation length is defined by competition between intermolecular coupling and disorder^19^, which can be clearly seen from the comparison between the PL spectra from high-quality and low-quality samples. In some 1L samples with low-quality growth, the FR exciton peak at ~680 nm was weak; instead, high-energy CT peaks appeared in the PL spectra (Fig. S12; Supplementary Information note 6). Compared with the high-quality 1L pentacene samples, as shown in Fig. 2, these low-quality 1L samples showed a broader peak and a lower PL peak intensity at 680 nm and longer radiative lifetimes of the FR excitons (Fig. S13), which was caused by the reduced coherence length in the low-quality 1L samples. In the experiment, we also found that the disorder and interfacial states increased as the sample thickness increased, which led to decoherence and reduced exciton delocalisation length in thick samples, such as 2L and bulk samples (both had J-aggregation). The 2L pentacene sample also showed a sharp emission peak at 680 nm, but the peak had a slightly larger width and a much lower PL intensity compared to 1L (Fig. S14). Additionally, bulk pentacene showed a broad PL emission, and the 680 nm emission peak was almost negligible (Fig. S8) due to the significantly reduced coherence length caused by the disorder and interfacial states in the sample, similar to other bulk organic thin films^33^. In comparison, the 1L PEN sample showed a very strong and narrow linewidth PL emission at 680 nm, which is a key characteristic of coherent emission. Thus, the lack of crystallinity, presence of more defect sites, inhomogeneous growth and high thickness compared to the 2D state lead to loss of coherent states in bulk thin film pentacene samples. Of course, our 1L samples were not perfect. There is still much room to improve the sample quality, which might give us a higher coherence length and a longer exciton diffusion length. Third, the quantum confinement of the system is beneficial to the speed of exciton transport since the confinement can increase the exciton oscillation strength^27^ (Supplementary information note 7). Our 1L pentacene samples (~1.2 nm thick) have intrinsically high confinement at the 2D quantum limit. Moreover, the excitons in 1L are highly anisotropic and can be aligned in a quasi-1D space along the *b* axis, which further increases the confinement of exciton transport in 1L pentacene. In summary, structural uniformity with a defect-free interface, highly confined excitons and excellent intermolecular coupling (large oscillator strengths) results in the supertransport of excitons in 1L pentacene. On the other hand, the diffusion coefficient (3.5 cm^2^/s; Fig. 3d) of the CT excitons in the WL sample was also much larger than that of other similar CT excitons reported recently^24^. Even though no coherence was observed in WL, the strong spatial confinement of the excitons (~0.6 nm thick) and the almost defect-free interfaces in WL samples led to long and fast propagation of long-lived CT excitons^21^. The high diffusion length obtained in WL PEN samples is critical for applications in high efficiency solar cells and charge separation^52^. In addition, waveguiding effects can be excluded, as the thicknesses of the 1L and WL pentacene samples were <1.5 nm and that of the h-BN layer underneath was only ~4 nm (measured by AFM), thus making them insufficient for any light trapping and waveguiding, similar to previous reports^22^.

## Discussion

It is important to remark here that the observed diffusion corresponded to bright singlet excitons in our 1L and WL pentacene samples and that the long-lived triplet excitons generated via the singlet fission in pentacene did not affect the diffusion measurements (Fig. 3). The reasons are twofold. First, the triplet states are non-radiative dark states^53,54^ and cannot be detected by the confocal photoluminescence setup we employed for our measurements^32^. Second, the regeneration of emissive singlets via subsequent triplet fusion is not allowed in pentacene, and singlet fission in pentacene is expected to be exothermic and unidirectional^55,56^ because the relaxed triplet in pentacene has significantly less than half the energy of the singlet. This is in contrast to the situation in tetracene, where the near-degenerate singlet and triplet-pair energy levels permit both singlet fission and triplet fusion to occur.

It is also important to remark here that the observed supertransport of excitons in 1L pentacene should be related to the unique properties of singlet fission in pentacene. First, singlet fission in pentacene, a fast non-radiative decay, occurs within a time scale of ~80 fs^56^. On the picosecond time scale, the singlet fission in pentacene thin films has a very small total yield of ~2%^40^. In our 1L pentacene sample, both our pump-probe (Fig. 2d) and TRPL (Fig. 2c inset) measurements showed a monoexponential radiative decay of ~4 ps, which indicates minimal contribution of non-radiative singlet fission mechanisms in the 1–10-ps regime, consistent with a previous report^40^. Second, our 1L pentacene sample at 77 K showed a much stronger PL intensity than our 2 L and bulk pentacene samples (Figs. 2b, S14 and S8), which suggests a highly reduced singlet fission rate in our 1L sample. This could be attributed to the minimised disorder in our 1L samples and the impact of molecule packing on singlet fission in organic molecules^57–60^. Of course, further exploring the aggregation and layer-dependent singlet fission in 2D pentacene samples would be a very interesting future topic.

### Superfast and angle-dependent transport of coherent excitons at room temperature

**Fig. 5:**
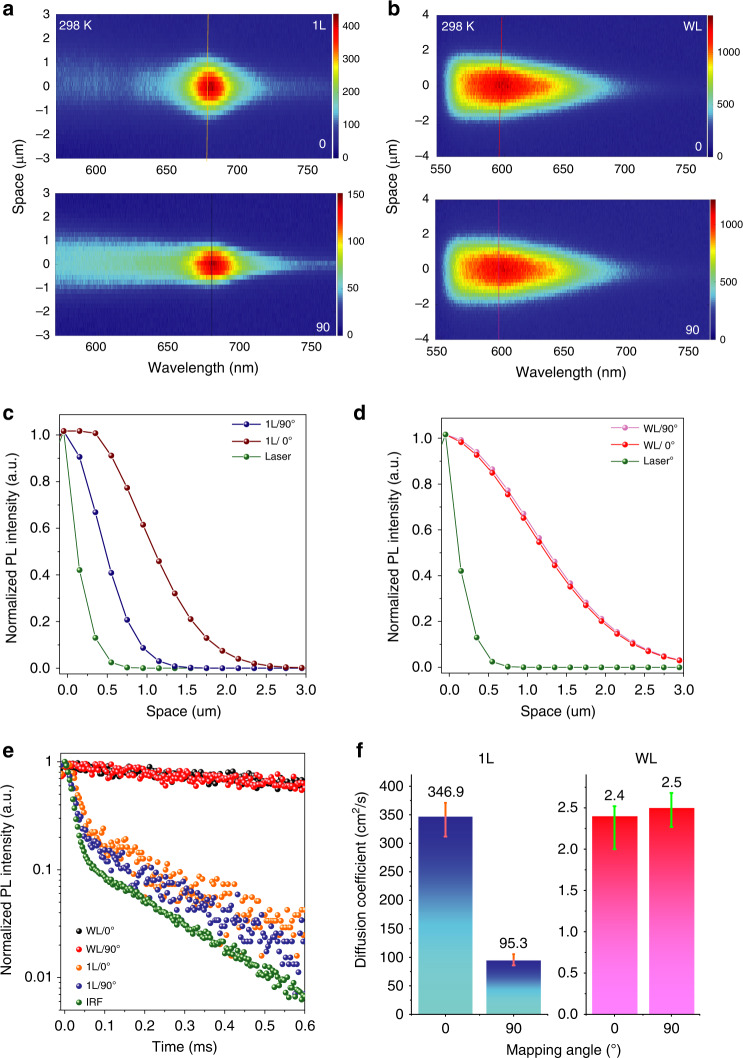
Superfast and angle-dependent exciton transport at room temperature. **a**, **b** Measured contour plots of PL intensity as a function of emission wavelength and space for 1L (**a**) and WL (**b**) pentacene samples at room temperature, with spatial mapping direction parallel (top panel; marked as 0°) and perpendicular (bottom panel; marked as 90°) to the *b* axis of the molecular lattices, as shown in Fig. S3a. In our measurement system, the mapping direction is parallel to the polarisation of the incident laser. The polarisation angles 0° and 90° have been marked according to the references set in the Fig. 2f inset. **c**, **d** Spatial profile plots along the dotted lines in **a**, **b** along two perpendicular mapping directions (0° and 90°), showing the angle-dependent exciton transport in 1L (**c**) and WL (**d**) pentacene samples. **e** Time-resolved PL emission (normalised) from WL and 1L pentacene samples at room temperature. The black and red balls represent the decay curves measured from WL pentacene samples with incident polarisation angles of 0° and 90°, respectively. Effective lifetimes of 2.8 and 2.8 ns were extracted from the black and red decay curves, respectively, by fitting with deconvolution of the instrument response function (IRF) (green dots). The orange and blue balls represent the decay curves measured from 1L pentacene samples with incident polarisation angles of 0° and 90°, respectively. The orange and blue decay curves were fitted with deconvolution of the IRF, giving effective short lifetime values of 12.7 and 5.6 ps, respectively. **f** Comparison plots of extracted diffusion coefficients measured from 1L (left panel) and WL (right panel) samples under the designated mapping angles at room temperature

Semiconductors with long-range and fast transport of excitons at room temperature are critical for future high-speed excitonic circuits and quantum computing devices^13–15^ that can operate at room temperature. Here, we also observed superfast and angle-dependent transport of excitons from 1L pentacene samples at room temperature (Fig. 5). In our measurement system, the polarisation angle of the incident laser was fixed to be parallel to the diffusion mapping direction. The exciton diffusion lengths and effective diffusion coefficients along two axes (*b* and *a*, labelled as 0° and 90°, respectively) of the pentacene unit cell were measured by rotating the sample with respect to the polarisation of the incident laser. The measured exciton diffusion length for the 1L pentacene sample showed a maximum value of 0.93 ± 0.09 μm along the *b* axis (0°) and a minimum value of 0.37 ± 0.02 μm along the *a* axis (90°; Fig. 5a, c). In contrast, the measured exciton diffusion length for the WL sample was not angle dependent, showing a constant value of ~1.31 ± 0.2 μm (Fig. 5b, d). The exciton emission lifetime measured from the 1L pentacene sample changed from ~12.7 ps at a polarisation angle of 0° to ~5.6 ps at a polarisation angle of 90° (Fig. 4e), whereas the lifetime measured from the WL pentacene sample remained largely unchanged (~2.8 ns) with changing incident polarisation angle (Fig. 5e). The effective exciton diffusion coefficients were thus extracted and are shown in Fig. 5f. The 1L pentacene sample showed clear anisotropic exciton transport, with a very high effective diffusion coefficient of 346.9 ± 24.1 cm^2^/s along the *b* axis and 95.3 ± 10.2 cm^2^/s along the *a* axis (Fig. 5f), whereas the WL sample showed a consistent effective diffusion coefficient of ~ 2.4 ± 0.1 cm^2^/s along different axes of the sample due to its isotropic excitonic emission behaviour.

In conclusion, in high-quality atomically thin organic 2D semiconductors, we observed supertransport of excitons between coherent excitonic states with a large effective diffusion coefficient of 346.9 cm^2^/s at room temperature, which is ~1 to several orders of magnitude higher than the values reported for other organic quasi-1D and thin film molecular aggregates^19–21^. We successfully identified molecular aggregation-sensitive PL emissions in atomically thin single-crystal organic semiconductors with different types of aggregation (H- and J-type) at the 2D quantum limit. The 1L pentacene samples with J-type aggregation (FR exciton dominated) showed superradiant emission, which was experimentally confirmed by the temperature-dependent PL emission, highly enhanced radiative decay rate, significantly narrowed PL peak width and strongly directional in-plane emission. This can be attributed to the constructive dipole coupling in the J-aggregates in 1L pentacene that forms an enhanced net optical dipole moment. The excitons in 1L pentacene samples were determined to be delocalised over ~135 molecules. In addition, the supertransport of excitons in monolayer pentacene samples showed highly anisotropic behaviour. On the other hand, the WL pentacene samples with H-type aggregation (CT exciton dominated) showed fast migration of isotropic CT excitons with a measured diffusion coefficient of ~ 2.4 cm^2^/s at room temperature, a few times larger than that reported for other similar CT excitons^24^. Even though no coherence was observed in WL, the strong spatial confinement of the excitons (~0.6 nm thick) and the almost defect-free interfaces in WL samples lead to long and fast propagation of long-lived CT excitons at the 2D quantum limit^21^. Our results provide an important experimental demonstration of the long-range and superfast transport of excitons between coherent states at the 2D quantum limit, paving the way for promising applications in future high-speed excitonic circuits, quantum computing devices, fast OLEDs, and other optoelectronic devices.

## Materials and methods

### Material growth

Few layer h-BN samples were prepared by mechanical cleavage method and placed onto a SiO_2_ layer which was thermally grown; 285 nm in thickness and deposited over a silicon substrate. The topology of the sample was examined by an optical microscope before physical vapour deposition (PVD) process. Pentacene samples (Chem Supply: P0030-1G) were then deposited over exfoliated h-BN which were placed in the centre of vacuum tube in furnace. The h-BN samples were then placed around 15 cm downstream in the vacuum tube, and a pressure of ~10^–4^ mbar was achieved in quartz tube using a molecular pump. The furnace temperature was increased to 130–150 °C for 15–30 mins to grow the pentacene samples. The furnace was then allowed to cool down naturally and vacuum was maintained. The number of layers were optimised by controlling the source temperature, growth time and substrate position. All the samples were characterised and the layer thickness was determined using atomic force microscope (AFM) measurements, in ambient room temperature conditions using a Bruker Multi-Mode III AFM instrument.

### Optical characterization

Horiba LabRAM system combined with a confocal microscope was used to conduct PL measurements at both room temperature and 77 K. A charge-coupled device (CCD) Si detector was used in conjunction with a 532-nm diode-pumped solid-state (DPSS) laser which was used to excite the samples. For low temperature (above and at 77 K) measurements, the samples were placed in a microscope-compatible chamber with a temperature controller (which used liquid nitrogen as the coolant). For polarisation dependent measurements the incident angle of polarisation of laser was controlled by a half-wave plate, and the angle of polarisation on the emission side (*θ*) was then determined using a polarizer which was angle-varied and placed in front of the detector. Time-resolved PL measurements were performed using a setup that incorporated μ-PL spectroscopy and a time-correlated single-photon counting (TCSPC) system. Pulsed laser which was linearly polarised (frequency doubled to 522 nm, with a 300-fs pulse width and a 20.8-MHz repetition rate) was channelled to a high numerical aperture (NA = 0.7) objective (Nikon S Plan 603). The PL signal was then gathered by a grating spectrometer, thus either collecting the PL spectrum through a charge-coupled device (CCD; Princeton Instruments, PIXIS) or detecting the PL intensity decay using a Si single-photon avalanche diode (SPAD) and the TCSPC (PicoHarp 300) system. The double exponential decay from 1L PEN is attributed to the biexponential decay in the instrument response function (IRF). The double exponential decay in the IRF is due to the delay in the response timings of the MPD SPAD® photon detector and the PicoHarp 300 coupled to it. A similar system response has been reported for an equivalent system^61^. Similar biexponential decays are commonly reported for such systems^62^. Thus, we used deconvolution to extract a monoexponential decay curve for 1L, and all the lifetime data results were fitted after deconvolution with the IRF (Fig. 2c). This monoexponential decay was further confirmed by our TR pump-probe femtosecond resolution measurements, which also clearly demonstrated a monoexponential decay for 1L PEN at 77 K.

### Measurement of exciton diffusion lengths

Exciton diffusion measurements were carried out using the same PIXIS CCD detector coupled with a ×100(NA = 1.49, oil suspended) objective lens. A pulsed 522-nm laser was used for excitation with a beam diameter of ~500 nm (confirmed by CCD imaging) and a collection time of 1 s per measurement. The diffusion mapping direction was well aligned with the polarisation of the excitation laser. The collected light was spectrally filtered to remove the pump laser wavelength. Spectral measurements were performed using a grating spectrometer (Acton, SpectraPro 2750). The focal plane of the sample was imaged using the zeroth order of the grating and the spectrometer CCD, giving a spatial resolution of ~200 × 200 nm in space, corresponding to a pixel (20 μm × 20 μm) on the CCD. The PL intensity at a particular excitonic emission energy was plotted as a function of the distance from the excitation centre (*x* = 0 in Figs. 3–4). The spatial extent of exciton diffusion (diffusion length *L*_*D*_) was extracted by fitting the experimental data and laser profile with a 1D Gaussian diffusion model^21,43^. See Supplementary Information note 8.

### Pump-probe transient reflectivity measurement

Transient reflectivity measurements were performed with a home-built pump-probe setup. Degenerate, linearly polarised pump and probe pulses were produced by splitting ~200 fs pulses from a non-collinear optical parametric amplifier (Light Conversion Orpheus-2N) tuned to 680 nm. A half-wave plate in the probe line rotated the polarisation to be orthogonal to that of the pump. The pump was delayed using a motorised delay stage to control the pump-probe timing. The pump and probe pulses were recombined to be nearly spatially overlapped and were focused onto the sample with a 25.4 mm focal length lens. Telescopes in the pump and probe lines were used to tune the divergence/waist size of the pump and probe beams. The focal spot size and fluence were 30 µm and 90 µJ/cm^2^ and were 15 µm and 12 µJ/cm^2^ for the pump and probe, respectively.

The probe pulses were spectrally resolved across a fast CMOS array detector (Andor Zyla) by an imaging spectrometer (Andor Kymera). The detector recorded spectra at 5.21 kHz, synced to the laser repetition rate of 20.83 kHz. A data analysis technique based on lock-in detection was used to isolate only the component of the probe modulated at the same frequency as the chopper in the pump beam path, which was also synced to the laser output. A polarizer was used to filter out the pump signal coupled with an iris. The residual pump signal still appeared minimally both as a background signal and as interference fringes between the pump and the probe. The interferometric pump contributions were removed using a Fourier filter with a width of 400 fs, and the static background was removed by subtracting the signal observed at negative pump-probe delays (i.e., the probe arriving before the pump). The data presented in Fig. 2 are the result of 11 and 5 repeats of a 15-ps scan of the pump-probe delay for 1L and WL, respectively, with a step size of 100 fs and a 1-s dwell time at each step.

### Spatial-temporal mapping

Spatial-temporal mapping was carried out on our samples at 77 K using a movable lens, focusing the PL signal onto the CCD detector with a movement range of 10 μm from the central position on the sample (see the schematic in Fig. 4a). The temporal decay maps for 1L and WL are consistent with the results shown in Fig. 3 of the main text. The data shown in Fig. S4b were smoothed in the post-processing by using one level of interpolation (image spline) between the recorded data from the system to extract the diffusion coefficient due to the limited number of data points in the regime.

## Supplementary Information

Supplementary Information
