# Supplementary material for: Supertransport of excitons in atomically thin organic semiconductors at the 2D quantum limit
Source: Light Sci Appl. 2020 Jul 6;9:116. doi: 10.1038/s41377-020-00347-y (PMC7338549; doi:10.1038/s41377-020-00347-y)
Supplement: Supplementary file 1 — Supplementary Information [file 41377_2020_347_MOESM1_ESM.pdf]

Supplementary Information for

Supertransport of Excitons in Atomically Thin Organic Semiconductors at the 2D Quantum Limit

Ankur Sharma,[1] Linglong Zhang,[1] Jonathan O. Tollerud[2], Miheng Dong,[1] Yi Zhu,[1] Robert Halbich,[1] Tobias Vogl,[3] Kun Liang,[1,4], Hieu T. Nguyen,[1] Fan Wang,[5] Shilpa Sanwlani[2], Stuart K. Earl[2], Daniel Macdonald,[1] Ping Koy Lam,[3] Jeffrey A Davis[2] and Yuerui Lu[1,3,4*]

[1]Research School of Electrical, Energy and Materials Engineering, College of Engineering and Computer Science, The Australian National University, Canberra, ACT, 2601, Australia

[2]Optical Sciences Centre, Swinburne University of Technology, Hawthorn, Victoria, 3122, Australia

[3]ARC Centre of Excellence for Future Low-Energy Electronics Technology, Australia

[4]Centre for Quantum Computation and Communication Technology, Department of Quantum Science, Research School of Physics and Engineering, The Australian National University, Acton ACT, 2601, Australia

[5]School of Mechatronical Engineering, Beijing Institute of Technology, Beijing 100081, China

[6]Institute for Biomedical Materials and Devices (IBMD), Faculty of Science, University of Technology Sydney, NSW 2007, Australia.

* To whom correspondence should be addressed: Yuerui Lu (yuerui.lu@anu.edu.au)

Supplementary Information Note 1: Exciton types and theory

Excitons are defined as the quasi bound state of electron and hole pair, which are attracted to each other by coulombic electrostatic forces. Based on the degree of charge separation which in turn is determined by material dielectric, the excitons can be broadly classified into the following categories[1]:

Wannier-Mott (W-MT) excitons are generally found in materials with large dielectrics, thus electric field screening reduces the Coulomb interaction between e^- and h^+. This results in much larger size for this kind of excitons, which is sometimes larger than space lattice. In W-MT excitons, smaller effective mass of e^- also favours the large radii of excitons. Typical binding energies reported here are around 0.01 eV. They are found mostly in inorganic semiconductors have been recently shown to exist in monolayer TMDs as well.

Frenkel (FR) excitons, whereas are found in materials with low dielectric constants, the Coulomb interaction between e^- and h^+ is much stronger, thus have smaller radii. They are observed mostly in organic materials such as anthracene, pentacene and other materials with low dielectric constants, thus enhancing the Coulomb interaction between e^- and h^+ resulting in much smaller radii. The size is of the same order as a unit cell and often reside on the same molecule. Binding energies range from 0.5-1.0 eV.

Charge transfer (CT) excitons are the intermediates between FR and W-MT excitons. They occur when e^- and h^+ occupy adjacent molecules. They are considered as a localized, structured version of W-MT exciton, where a higher dielectric constant permits the radius to expand over many sites. Their excitonic wave function resembles that of a hydrogen atom. Both CT and FR excitons can have localized wave functions.

Supplementary Information Note 2: Role of molecular packing and temperature on optical properties

The effect of molecular packing on excitonic states was firstly reported by Kasha *et al.*[2], who extended the geometry-related FR exciton treatment by Davydov.[3] In our model, we define J to be excitonic coupling between different local FR exciton states located at nearest-neighbour molecules. The oscillator strength of an optical transition is a function of the total TDMs in an aggregate of molecules as shown below theoretically. If the Coulombic repulsion is stronger than the Coulombic attraction, their competition will favour a positive J value ($J > 0$) and vanishingly small total TDM, which will lead to an optically forbidden lowest excited state (S_1) (Figure S3b)[4]. In contrast, if the Coulombic attraction is stronger than the Coulombic repulsion, their competition will favour a negative J value ($J < 0$) and large total TDM, which will result in an optically allowed S_1 (Figure S3c). The result of this competition mainly depends on the orientation rather than the distance between molecules.[5] Molecular aggregates are usually considered to be H-type when $J > 0$ and J-type when $J < 0$.[5] Generally, side-by-side molecular orientations lead to $J > 0$, whereas head-to-tail orientations lead to $J < 0$.

Based on our simulation results, the total TDM and oscillator strength for the lowest excited states (S1) in WL were both zero, which led to non-luminescent S1 states at a temperature of absolute zero. In contrast, the total TDM for S1 in 1L had a large magnitude with the direction along the b axis of its unit cell, and its corresponding oscillator strength for S1 was also non-zero. This resulted in luminescent S1 states at absolute zero. The large magnitude of net TDM corresponds the phase-locking and constructive coupling of TDMs in 1L pentacene cooperatively resulting in sharply enhanced PL emission, which is defined as superradiance. This is supported by our temperature dependent PL measurements shown in Figure 2. At low temperature the emission from 1L is much stronger and sharper as compared to WL, which emission reduces as the temperature decreases to 77 K from room temperature. However, thermal disorder with increasing temperatures will cause fluctuations for both onsite energies and excitonic couplings and accordingly break the symmetry in the aggregation model. This will lead to thermal deactivation and activation for PL emissions in J- and H-type aggregations

in 1L and WL pentacene, respectively. This results in decreased emission of PL in 1L at room temperature and an enhanced PL emission from WL.

For a transition from state $|\Psi_a\rangle$ to another state $|\Psi_b\rangle$, its transition dipole moment (TDM) is[6]

$$\mathrm{TDM}(\mathrm{a} \rightarrow \mathrm{b}) = \langle \Psi_a | \hat{\mu} | \Psi_b \rangle = \langle \Psi_a | q\boldsymbol{r} | \Psi_b \rangle = \int \Psi_a^*(\boldsymbol{r}) q(\boldsymbol{r}) \boldsymbol{r} \Psi_b(\boldsymbol{r}) d\boldsymbol{r} \quad (1)$$

where *q(r)* is the charge on the particle at an arbitrary position '*r*'. TDM is not the oscillator strength *(f)*, but it is the most crucial parameter for determining *f*[7].

$$f(\mathrm{a} \rightarrow \mathrm{b}) = \frac{2m_e(E_b - E_a)}{3\hbar^2} \mathrm{TDM}(\mathrm{a} \rightarrow \mathrm{b})^2 \quad (2)$$

where, E_b and E_a are the energy levels associated with states '*b*' and '*a*' respectively.
In short, the magnitude of TDM determines the strength of the optical transition to a large extent. Additionally, its α (= *x, y, z* or *a, b, c*) component determines the incident light polarized absorption (or transition) along α direction.

Supplementary Information Note 3: Coherence number determination and effect of vibration and phononic interaction

The high crystallinity obtained in 1L pentacene samples will result in large coherence lengths and high diffusion coefficients but the effect of vibrations or phonon interaction in the lattice cannot be ignored. The phonon or vibrational coupling affects the PL spectra which is characterized by a spectral phononic sideband emission in addition to the sharp 0-0 Frank Condon emission, which is at 680 nm in our case (See Main Text Fig 2). As suggested by Zhao *et al.*[8] and Tanaka *et al.*[9], the coherence number will start to decrease as the vibrations increase

and hence resulting in lower diffusion lengths and diffusion coefficients.

To quantify the phonon interaction we have followed the technique reported by Spano *et al.*[10] to precisely determine the coherence number (N_c) in 1L pentacene at 298 K and 77 K. In effect, this vibration serves as a probe for the coherence length. When the optical transition from the lowest exciton to the vibration less electronic ground state (the "0−0" transition) is symmetry-allowed, as in a J-aggregate, the ratio of the 0−0 to 0−1 transition line strengths can be used to determine the coherence number. The coherence number can be very precisely number determined taking into account the spectral line strengths (integrated PL) of the coherent peak and the sideband emissions by the following equation:

$$\frac{N_c}{\lambda^2} = \frac{I_{0-0}}{I_{0-1}}$$

Here, the I_{0-0} is referred to the FR emission at 680 nm and I_{0-1} is the phonon sideband emission caused due to phonon or vibrations in the lattice. N_c is the coherence number and λ^2 is the Huang-Rhys factor in pentacene. At 298 K and 77 K the PL spectra can be fit by two Lorentzian peaks as shown in Figure S6a and S6b respectively.

The Huang-Rhys factor used for our calculation is taken to be 0.574 which has been theoretically determined for pentacene and taken from ref[11]. The extracted values of spectral strengths and 0-0 and 0-1 transitions and subsequent N_c has been tabulated as below.

**Temperature (K)**	**I_{0-0} (a.u.)**	**I_{0-1} (a.u.)**	**N_c**
298	84858	1615	30
77	287336	1214	135

The value of N_c derived at low temperature is much higher than previously reported values from thin film pentacene and similar oligoacene films like tetracene (N_c= 10), thus confirming the limited effect of vibrational or phononic coupling in high crystalline 1L PEN samples specially at low temperatures. The high Value of N_c directly corresponds to higher diffusion lengths and higher diffusion coefficients in 1L PEN. The value of coherence number reported is an order of magnitude higher than previously reported values from thin film organic semiconductors and hence a high value of diffusion coefficient is expected from such a system operating with large coherent regimes to allow for excitons to transport quickly.

Supplementary Information Note 4: Excitonic character in WL and 1L governed by molecular packing

The excitonic nature of organic crystals plays an important role in determining their optoelectronic properties.[4,10,12] In organic semiconductors FR and CT excitons are most commonly observed.[13,14] (See Supplementary Information Note 1). As soon as the energy difference between CT and FR excitons becomes small, both excitons can interact and form new FR–CT mixed states.[14] Intermolecular coupling also influences the complex excitonic character in organic molecules. The mixed states are observed in most bulk and thin film organic semiconductors due to the lack of high crystallinity, scattering and the presence of disorders and interfacial states.[15-17] Precise and controlled growth of organic molecular crystals in atomically thin 2D states provides an ideal platform for isolating the smaller and tightly bound FR[18] excitons as they have closer packing of molecules within a unit cell.[18-20] In order to reduce the FR-CT mixing and to observe the aggregation-controlled coherence phase locking and subsequent superradiant emission, the crystallinity and molecular aggregation into H- (CT exciton dominated aggregation) and J-type (FR exciton dominated molecular aggregation) has to be controlled, as theoretically predicted by Spano *et al.*[10]

In our case, the highly clean and sharp crystalline system allowed us to observe long-range coherent supertransport from FR excitons which are de-localized over hundreds of pentacene molecules, due to of the minimized defect, disorders and interfacial states in the monolayer regime. WL pentacene samples, which had H-type molecular aggregation, showed in-plane isotropic emissions that were dominated by CT excitons. In contrast, 1L pentacene samples, which had J-type molecular aggregation, showed one sharp and strongly anisotropic PL emission peak (See Supplementary Information Note 2 for theoretical simulation details). The WL region showed much stronger PL emissions than the 1L regions at room temperature (Figure 1d). Compared with inorganic TMD 2D semiconductors, the WL shows a much broader PL spectra at room temperature, which is due to the strong couplings between FR and CT excitons in organic materials.[18,21] The broad PL spectrum is associated with various band energy levels formed in pentacene due to vibronic coupling between FR and CT states[10]. 1L on the other hand shows a single peak centred around 680 nm which is predominantly FR excitonic emission as explained by our polarization dependent measurements in Figure 2g and also explained below. We grew several samples of pentacene in various growth conditions, giving us different optical properties from 1L depending on growth conditions (Figure S3). The poorly grown 1L samples also gave us broad high energy CT excitonic peaks in addition to the narrow FR excitonic peak at 680 nm overlapping with the emission energies from WL in certain samples, showing the mixed FR and CT emissions from 1L. Whereas, some 1L samples gave us only the CT emission with little FR emission. This clearly highlights the role of well-defined molecular arrangements in de-coupling the intermixing of FR and CT excitons. We have chosen the 1L sample which gave us only FR emission for further analysis to independently study the behaviour of CT and FR excitons in WL and 1L *viz*.

We also performed PL measurements with an angle-resolved emission polarization (Figure 2f) to confirm the anisotropic nature of superradiant FR excitonic emissions from 1L. In the experiment, the incident polarization angle was controlled by an angle-variable half-wave plate and was fixed, and the polarization angle of the emission (θ) was determined by using an angle-variable polarizer located in front of the detector. In polarization-dependent PL measurement at 77 K, we found that intensity of PL emission from 1L strongly depended on the emission

polarization angle θ and shows a period of 90 degrees. On the contrary the emission from WL was completely anisotropic (Figure 2f). The strongly directional total TDM in 1L pentacene predicted by our simulation (Figure S3a) was expected to lead to an in-plane anisotropic emission of those FR excitons. The observed strong anisotropy of PL emission from 1L pentacene further confirms the strong optical alignment of molecules in a specific orientation and agrees very well with the prediction from our simulations, which confirms that the superradiant emission from 1L pentacene observed in 1L and 2L pentacene at low temperature is dominated by FR excitons.

Supplementary Information Note 5: Coherent exciton transport and supertransport

The diffusion of FR excitons that are commonly found in organic semiconductors migrate much slower as compared to their Wannier counterparts in inorganic materials. This is because of lower dielectric constant in organic materials and results in lower diffusion lengths in organic materials.[22,23] The energy transfer is dominated by incoherent Förster resonant energy transfer mechanism (FRET)[24], which involved hopping of charges from one chromophore to the other *via* dipole moment interactions. This mechanism is based on dipole-dipole electromagnetic interaction and occurs when emission spectrum of donor molecule has significant overlap with absorption of the acceptor molecule. It is a non-radiative transfer and relies on long-range Columbic coupling between the nearby molecules.[23] Due to this incoherent hopping, the diffusion of FR excitons is limited. Incoherent energy transfer is generally determined my inter-molecular spacing, defect states, excitonic lifetime and range of columbic coupling also referred to as Förster radius.[22,25] All these factors lead to smaller diffusion of FR excitons inorganic molecules and molecular assemblies.

However, in organic molecules the inter-molecular coupling can become very strong if the crystallinity is strong.[26,27] If the coupling between organic molecules is strong, the FR excitons can be delocalized over multiple molecules forming a delocalized coherent state for the exciton, which eventually leads to superradiant emissions.[28] In this phenomenon interactions between transition dipoles of individual molecules allow coherent delocalization across multiple sites. The corelated states lead to a macroscopic optical polarization proportional to the number of atoms or molecules comprising the coherent domain.[29,30] This leads to a net enhancement in the optical transitional dipole moment (TDM) value and sharp enhancement of the excitonic radiative decay rate of an ensemble of N_c independent emitters as compared to the radiation decay rate of a single emitter.[31,32] The same principle of coherent delocalized superradiant emission gives rise to an analogous phenomenon called cooperative energy transfer or supertransfer (ST).[33] The resulting enhanced oscillator strength from delocalization over large molecular assemblies can lead to large scale exciton transport. Here, the molecular assemblies

(consisting of N_c molecules) with comparable net TDMs can play the role of acceptors like individual molecules in FRET mechanism.[24] Thus, the excitation (exciton) can transfer to much longer distances in a coherent delocalized molecular assembly before annihilation, resulting in large values of diffusion coefficients. This strong intermolecular coupling plays an important role in developing coherent effects and determines long-range coherent exciton transport, which has been demonstrated in some quasi 1D systems recently. Delocalized excitons principally accelerate energy transfer as compared to incoherent hopping transfer because delocalization allows for a hopping length that is much larger as compared to intermolecular spacing.[25]

Supplementary Information Note 6: Relationship between crystalline order and diffusion length

As discussed above the charge or energy transfer mechanism in organic molecules requires hopping of excitation from one molecule to other (incoherent) or from one delocalized state to other (coherent). In either case, the distance between interacting molecules or states is critical. This intermolecular distance is a function of degree of crystallinity in the molecule.[34] Hence, the diffusion of excitons in organic system is a direct function of crystallinity. Lunt *et al*.[35] and Sim *et al*.[34] recently experimentally demonstrated that exciton transport diffusion length is a monotonic function of the extent of crystalline order. The reduction in diffusion lengths with decreasing crystallinity is due to increase in non-radiative losses in highly disorders systems, as determined by fluorescence quantum yield measurements as a function of grain size in Perylenetetracarboxylic dianhydride (PTCDA).[36]

We observed a similar trend during the growth optimization process of our pentacene samples. The samples which used h-BN surfaces that were exposed to air for a long time or had uneven morphology (detected optically) gave us low crystalline growth, as characterized by AFM measurements. In contrast the samples grown on homogenous and unexposed h-BN substrates gave a much stronger crystallinity and smooth pentacene growth, confirmed by surface roughness measurements. All the samples were characterized by AFM right after their growth. During the growth of pentacene samples in our case, we observed that the optical performance was also critically affected by the order of crystallinity in our growth as shown in Figure S5. The samples that had lower crystallinity showed a PL spectrum similar to earlier reports.[37] The broad PL spectrum obtained even from 1L samples is due to vibronic mixing of CT and FR states (Samples 2-4 in Figure S5). After several rounds of optimization, we could precisely control the growth conditions and orientation of molecules in 1L (Sample 1 in Figure S5). There were no CT peaks reported from highly crystalline samples, demonstrating only a sharp FR excitonic emission at 680 nm. Due to reduced defects, lack of interfacial sites and high crystallinity in our samples we could observe higher diffusion coefficients even for short-lived FR excitonic emissions from 1L pentacene samples.

Supplementary Information Note 7: Dimensionality and oscillator strength

Apart from coherent delocalization and high order crystallinity, the exciton diffusion process in organic molecules is also a function of the oscillator strength of the excitons.[23,26,27,38] The oscillator strength in return is strongly influenced by the effective dimensionality of the solid-state system as theoretically demonstrated by Wu *et al*.[39] The oscillator strength can be highly enhanced by reducing the effective dimensionality of the system. Thus, it is critical for us to develop a true 2D system that can confine the excitons in 2D quantum limit to further enhance the diffusion of the FR excitons.

The quantum confinement effect plays a key role in determining the spatial diffusion of excitons.[27] For the same reason, several recent attempts to achieve higher diffusion coefficients with molecular aggregates have used quasi-1D structures like tubes[25] or wires[27] to enhance the diffusion using J-type aggregation. In our pentacene WL and 1L system, the excitons were spatially confined in a 2D space, which increases the possibility of directed excitonic energy transfer within the 2D plane with limited out of plane losses and hopping. Further, in 1L as we have established the excitons were found to be anisotropic and aligned along the '*b*' axis of the unit cell of pentacene (Figure 2f in main text and associated text). This led to further confinement and transport if excitons are in a quasi-1D system, which eventually led to observation of ultra-high exciton diffusion coefficients in our samples.

Supplementary Information Note 8: Diffusion Length using direct CCD imaging

A robust method of determining exciton diffusion is in steady state which involves modelling of the spread of excitons in a 1-D model. In the limit of low exciton density, the number of excitons n (x, t) as a function of both time and position can be described from the diffusion equation.

$$\frac{\partial n(x,t)}{\partial t} = \frac{n(x,t)}{-\tau} + D\frac{\partial^2 n(x,t)}{\partial x^2} + pI(x)$$

where, D is the coefficient of diffusion, is the exciton lifetime measured separately, p is the probability of absorption and I(x) is the spatial profile of excitation. If we assume a Gaussian profile with a standard deviation of σ (or full width half maximum-FWHM) , the solution will lead to the following equation:

$$n(x,t) \propto \frac{1}{(2\sigma_0^2 + 4Dt)^{1/2}} \exp\left(\frac{-x^2}{(2\sigma_0^2 + 4Dt)}\right) \exp\left(\frac{-t}{\tau}\right)$$

In our measurements in Figure 3 and 4, the profile measured is a steady state profile unlike in measurements in Figure S12 where is a function of time. This steady-state profile is modelled by numerically integrating the above equation over time. This modelled profile is the used to fit the experimental data from Figure 3a, b to extract the diffusion length or s. We have then also fit the excitation laser Gaussian profile using the same fitting function to extract its width.

Both emission and laser profiles were fitted using the 1D Gaussian distribution model and their FWHMs were extracted. The spatial extent of exciton diffusion (diffusion length L_D) was extracted by taking the difference of these extracted widths from the fitting.

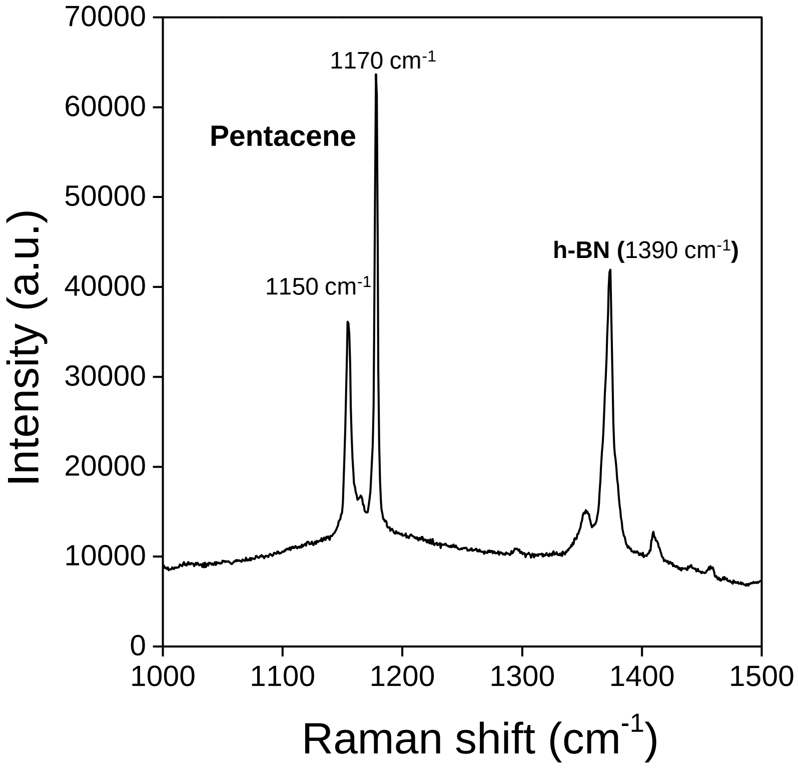

Figure S1 | Measured Raman spectrum from a 2D pentacene sample grown on a h-BN flake. Peaks at 1150 cm^{-1} and 1170 cm^{-1} are for pentacene and peak at 1390 cm^{-1} is for h-BN.

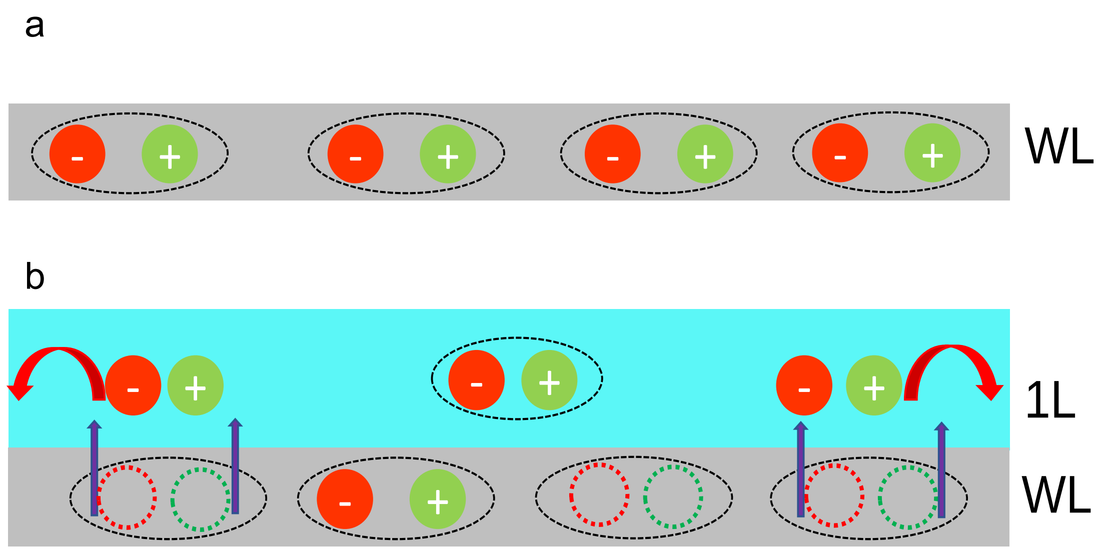

Figure S2 | Schematic diagram showing the interlayer charge transfer and different intra-layer charge transport mechanism in WL (a) and 1L+WL (b). As shown in a previous report[37], WL pentacene is not conducting due to the absence of intralayer π-π stacking. The charges interact by long range coulombic coupling in WL; in contrast 1L shows 2D hopping transport. The purple arrows represent interlayer charge transfer. The red curved arrows represent the hopping of charges in 1L. Here, we only show the photo-excited charges generated in WL, not in 1L.

The charges interacted by long-range Coulombic coupling in WL (Figure S2a); in contrast, 1L showed 2D hopping transport. The photo-excited charges in the 1L layers had much higher in-plane diffusion than those in WL, which led to lower PL efficiencies in 1L pentacene. For the 1L+WL hetero-structure (1L region), the photo-excited charges in WL underneath could be quickly transferred to 1L and diffused laterally in 1L before radiative emission occurred (Figure S2b). This led to quenching of the PL intensity. This type of ultrafast interlayer charge transfer phenomena has been observed for both inorganic 2D hetero-structures[40] and organic–inorganic interfaces.[41]

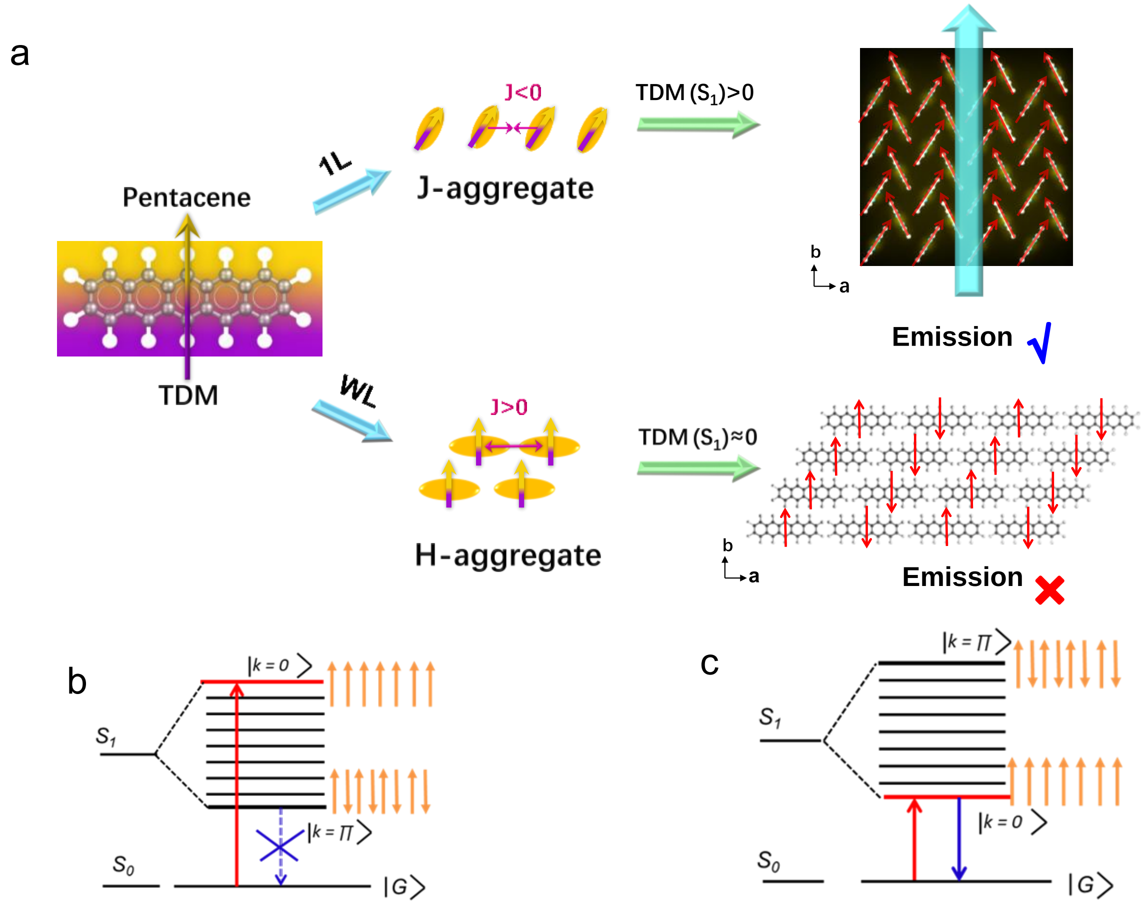

Figure S3 | Molecular packing and the FR exciton model. a, The TDMs at each molecule (red arrows) follow a head-to-tail packing in 1L as shown, while in WL TDMs are packed side-by-side to each other. The pink arrows represent the Coulombic interaction force between neighboring molecules. *J* is the excitonic coupling between different local FR exciton states located at nearest-neighbor molecules. Based on our simulation, *J* is negative in 1L (corresponding to J-type aggregation) and hence there is a net positive total TDM value along the "*b*" axis (as shown by purple arrow) allowing for emission from 1L, while *J* is positive in WL (corresponding to H-type aggregation) and hence a close to null total TDM, resulting in very little emission from WL. **b, c** Schematics showing the excited states in an ideal H- (a) and J-type (b) monomer. Optical transitions from and to ground state are only allowed for excited state band with k = 0, where k is the quantum number of the wave vector, representing the excitonic states. Orange arrows indicate the spin of electrons in each state. In J-type the 0-0 transition results in enhanced coherent emission due to coupling of TDMs, eventually causing super radiant emissions.

Reason for blue-shifted WL PL spectra: The WL is blue-shifted because it is an H-aggregate as compared with the spectra from 1L/2L. This can be understood by looking at the schematic in Figure S3b (See below), where the optically allowed bands are always at much higher energies as compared to the J-type aggregates. This blue-shift from H-aggregates was also predicted by Kasha *et al.*[2] and Spano *et al.*[9]

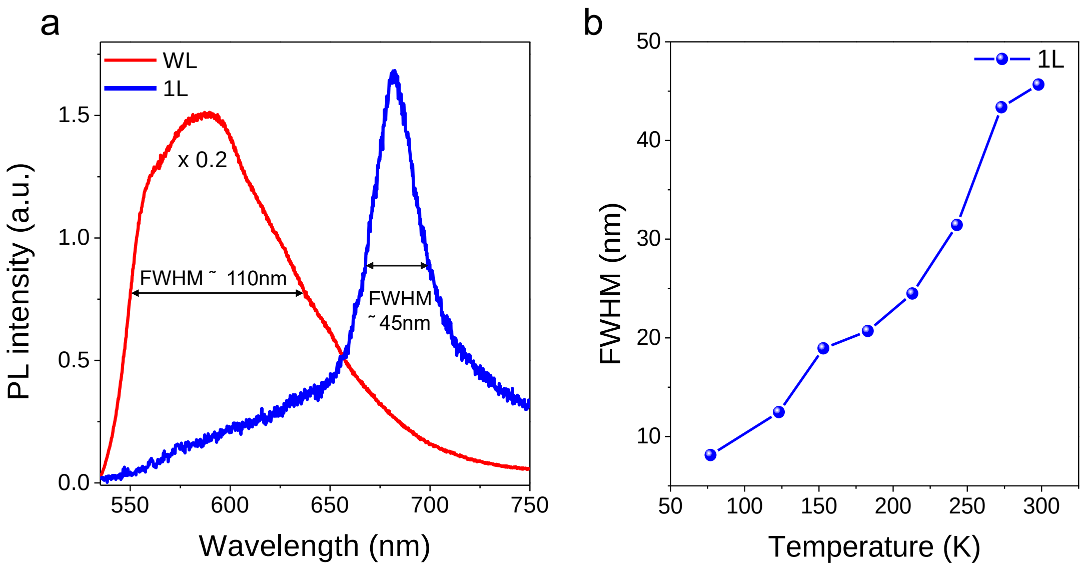

Figure S4 | Measured FWHM with decreasing temperature. a, PL spectra and respective FWHM at 298 K from WL and 1L**. b,** Measured Full-width half maximum (FWHM) as a function of temperature from 1L PEN.

The FWHM from WL overall is much higher at 298 K as compared with narrow linewidth from 1L, suggesting a dominant coherent component in the emission. As shown in the figure above the WL spectra is very board with FWHM ~110 nm, which arises due to CT and FR mixing in the WL. The FWHM from 1L has been plotted as a comparison show the extent of reduction in 1L as compared to broad WL PL spectra.

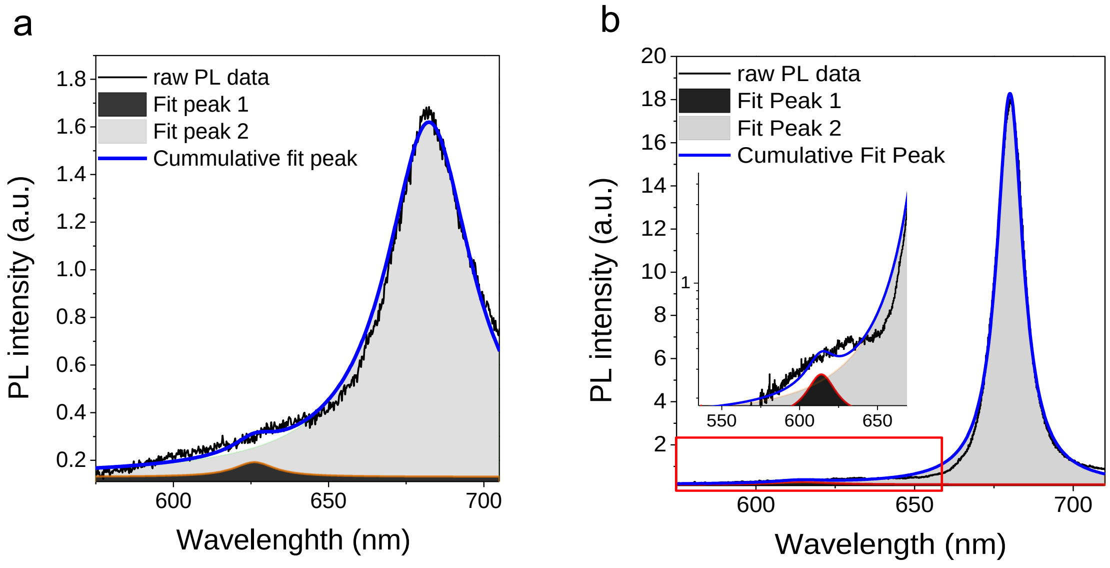

Figure S5 | PL spectra line strengths from 1L PEN. **a**, PL spectra as measured at 298 K. The shaded areas under the curve after a Lorentzian fitting function have been used to fit the PL data. **b**, PL spectra as measured from 1L PEN at 77 K fitted using two Lorentzian peaks. The inset shows a zoomed in area marked in red square on a log scale.

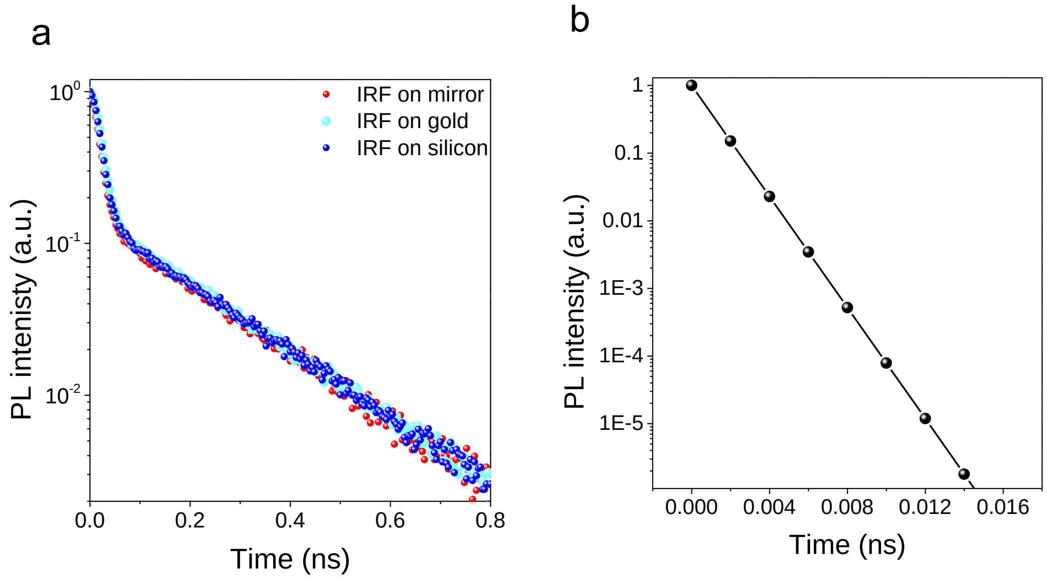

Figure S6 | Instrument response functions. **a**, as measured from different reflective surfaces. The IRF is very stable from different surfaces. **b**, Deconvoluting of one IRF over other gives a system resolution of 2.1 ps as shown by the deconvoluted graph.

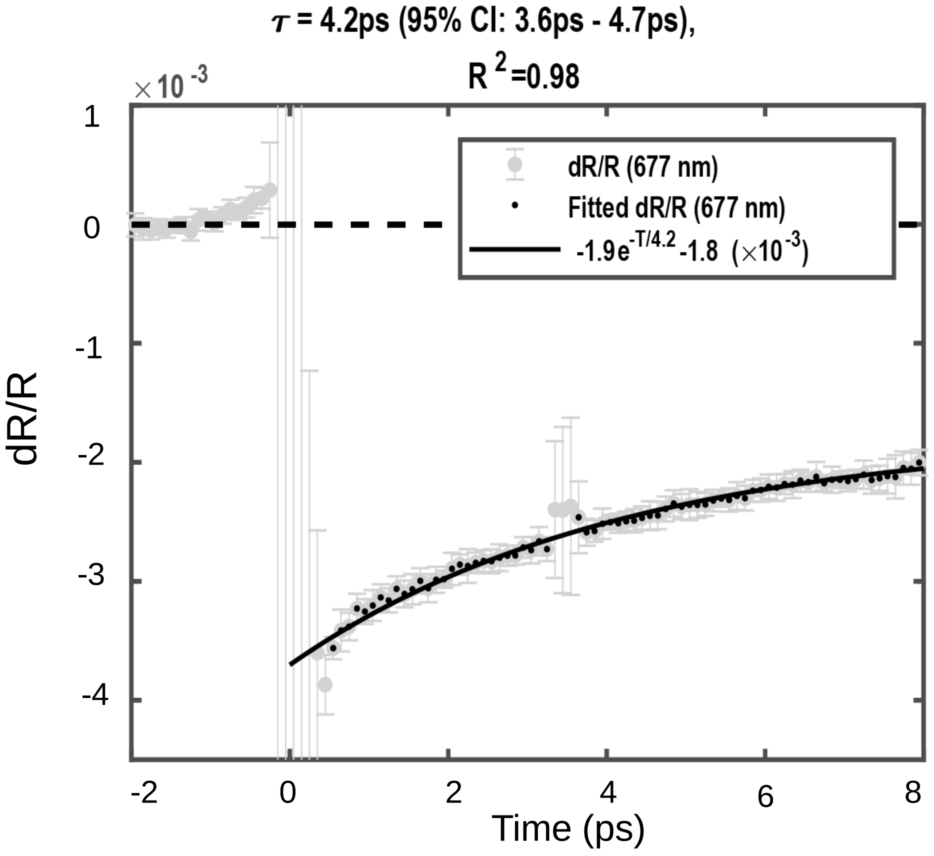

Figure S7 | Fitting and error analysis. The figure shows the fitting of the decay curve from pump-probe measurements with a 95% confidence interval fiving a lifetime range of 3.55-4.6 ps and the best fit with a value of 4.2 ps.

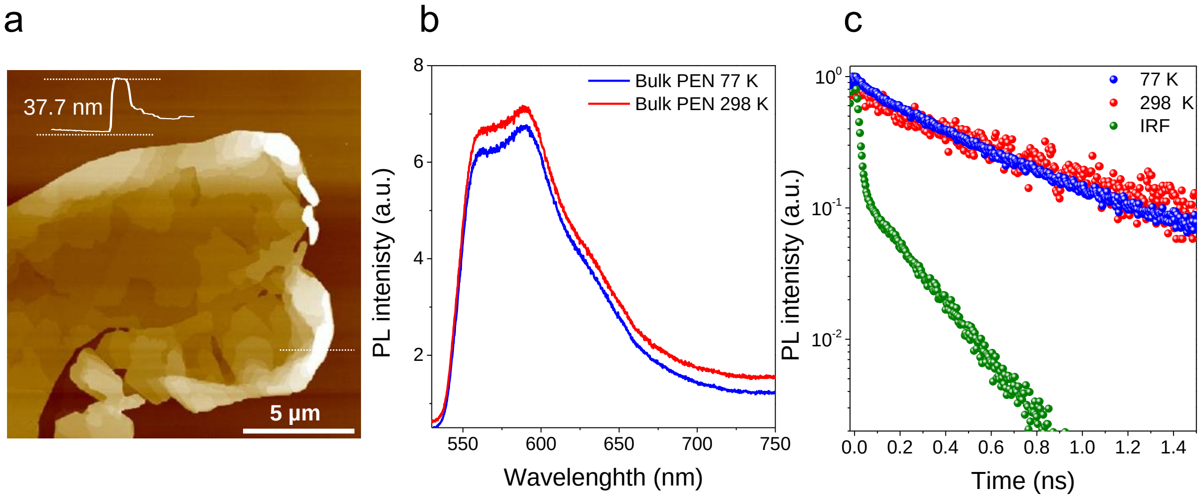

Figure S8 | PL emission from bulk thin-film pentacene, a, AFM image of the bulk thin film pentacene grown over h-BN with a thickness of around 37.7 nm. **b**, PL emission at 77 K from the bulk pentacene, which is dark as compared to WL. **c**, the decay curve from bulk pentacene at 298/77 K, giving an effective lifetime of 1.241/1.014 ns.

The emission from bulk pentacene layers is a broad PL spectrum, which changes negligibly with lowering of the temperature. The sharp peak emission at 680 nm is missing from the bulk pentacene. The broad PL spectra can be attributed to the coupling of CT and FR states, arising from the disorders and interfacial states, which has been reported in literature previously as well.[10,12] The lifetime from the bulk pentacene peak at 600 nm emission is 1.241 ns at 298 K, which changes to 1.014 ns at 77 K. The linewidth of the spectra is one order of magnitude higher (>100 nm as compared to 8 nm from 1L pentacene) from bulk pentacene, suggesting a non-coherent emission.

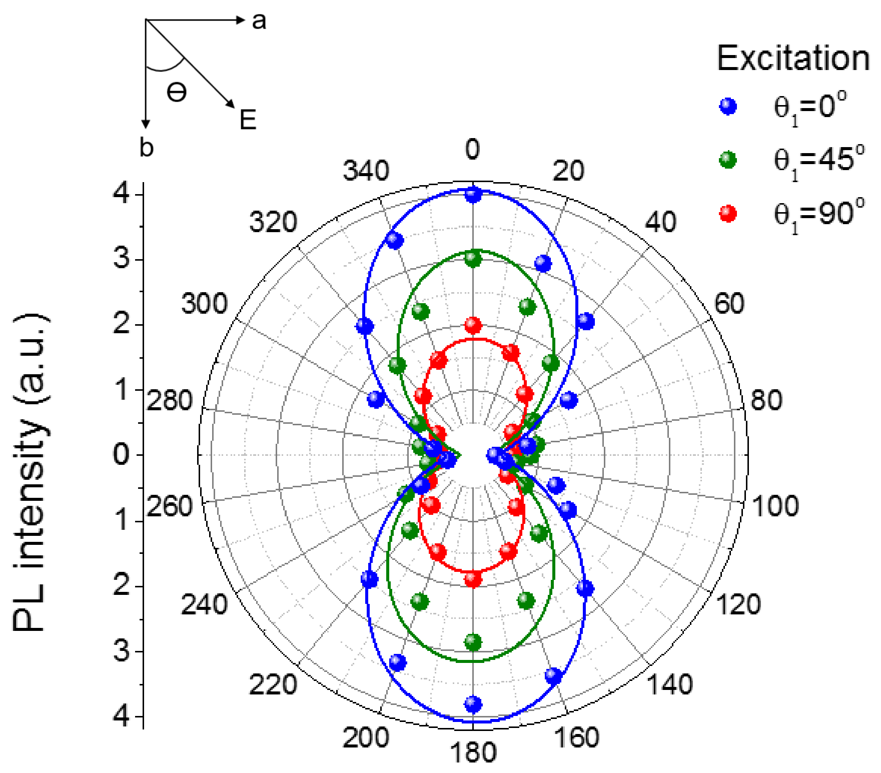

Figure S9 | Emission polarization measurements from 1L PEN at different laser polarizations. The polarization dependent PL measurements from 1L at 77 K. In experiment, the excitation polarization angle θ_1 was fixed at 0° and the polarization angle of the emission (θ_2) was determined by using an angle-variable polarizer located in front of the detector. We also changed θ_1 and repeated the measurements. We observed that PL emission from 1L always shows the maximum PL intensity at θ_2 = 0° and the minimum PL intensity at θ_2 = 90°, regardless of the excitation polarization angle θ1 used 0° (grey), 45° (magenta) and 90° (blue).

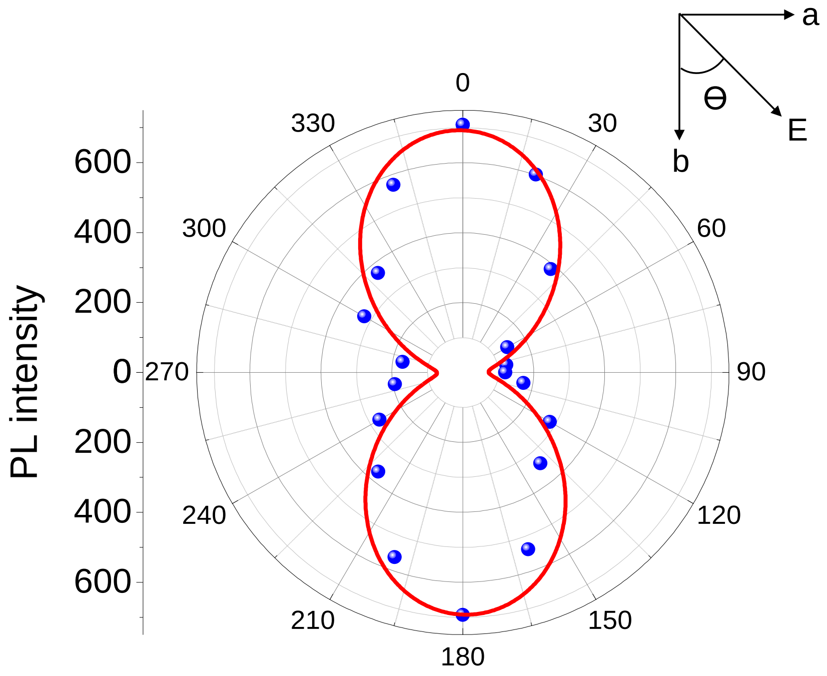

Figure S10 | Measured angle-resolved PL for excitation polarization from the 1L at room temperature. a, Measured angle-resolved PL spectra for emission polarization from 1L pentacene. The LDR is around 5.9 at room temperature. The incident polarization angle (θ) was controlled by an angle-variable polarizer. The excitation laser power remained constant. The solid balls represent the measured experimental values and solid lines are fitted curves using a $cos^2\theta$ function.

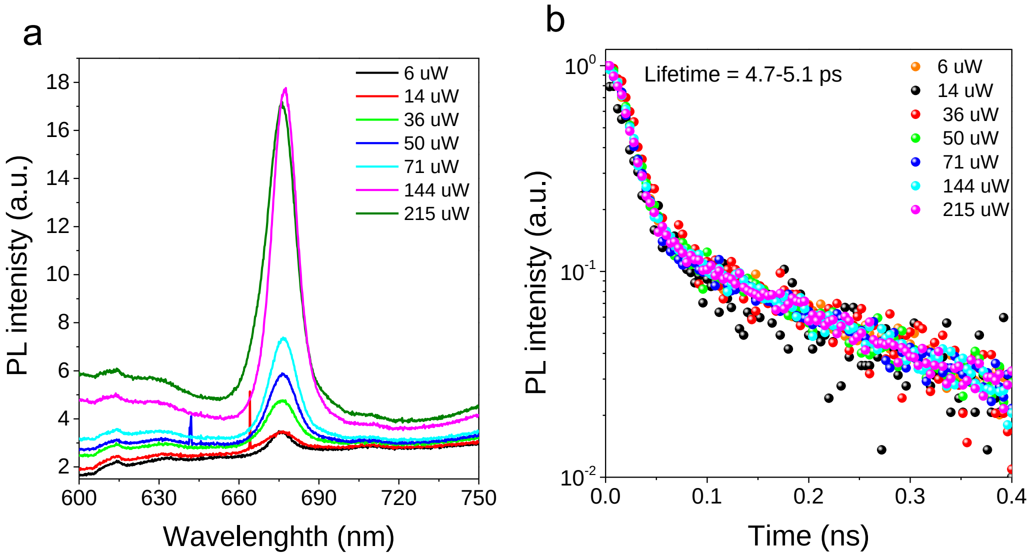

Figure S11 | Power dependent time-resolved PL at 77 K. Power dependent measurements at 77 K were carried out on 1L pentacene samples, to confirm the exciton-exciton annihilation effects. We observed an almost constant lifetime (4.7 -5.1 ps) ranging from a very low lase power to a high laser power ranging from 6 μW to 215 μW. The consistent lifetime suggest that the EEA does not play a key role in defining the exciton diffusion characteristics in our sample as shown in the figure below.

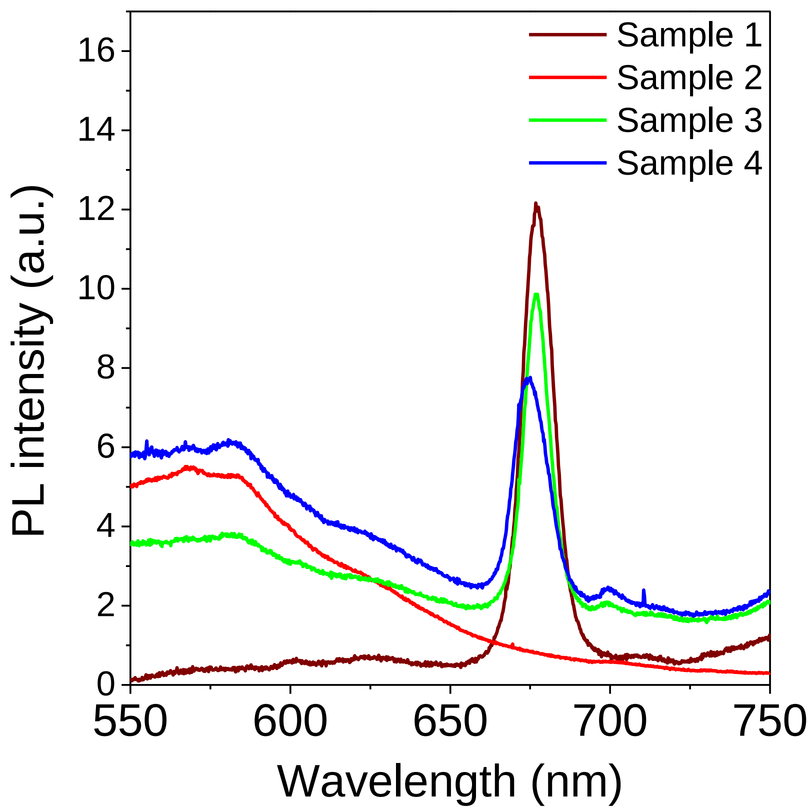

Figure S12 | Measured PL spectra from various quality of 1L pentacene samples at 77 K, fabricated under different growth conditions by CVD method.

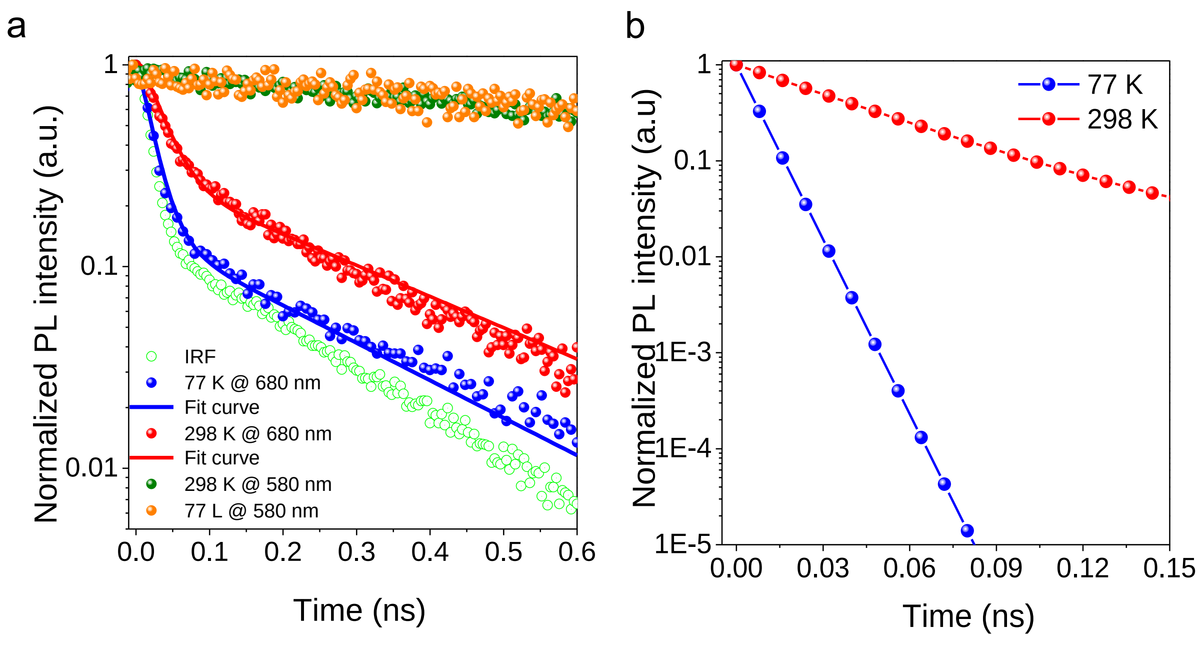

Figure S13 | Temperature dependent time-resolved PL for low-quality 1L pentacene (sample 3). a, Time-resolved PL emission (normalized) from 1L pentacene (680 nm, superradiant peak) sample 3 (See Figure S4) at 77 K (blue balls) and 298 K (orange balls). The solid lines represent the fitting curves for both the temperatures. An effective long lifetime of 51.2 ps was extracted from the orange decay curve (298 K) by a fitting with deconvolution using the instrument response function (IRF) (green dots). The lifetime extracted from blue curve (77 K) was 7.6 ps. The drastic reduction in lifetime with decreasing temperature, further supports the superradiance emission from 1L. The effective lifetime at 77 K is slightly higher than what we have reported in Figure 4 (4.1 ps), which may be due to the low crystallinity of the sample 3 as compared to sample 1. **b**, Time-resolved PL emission from the CT excitonic emission at ~580 nm from 1L pentacene sample. The lifetime extracted from the CT peaks is ~2.1 ns and 2.3 ns at 77 K and 298 K respectively. The lifetime is significantly higher as compared to the coherent FR emission peak at 680 nm, due to non-coherent emission from CT excitonic states, which do not show superradiant emission, further supporting the coherent emission visible at 680 nm even from low crystalline samples. **c**, Deconvoluted lifetime curves showing a drastic reduction in lifetime with decreasing temperature.

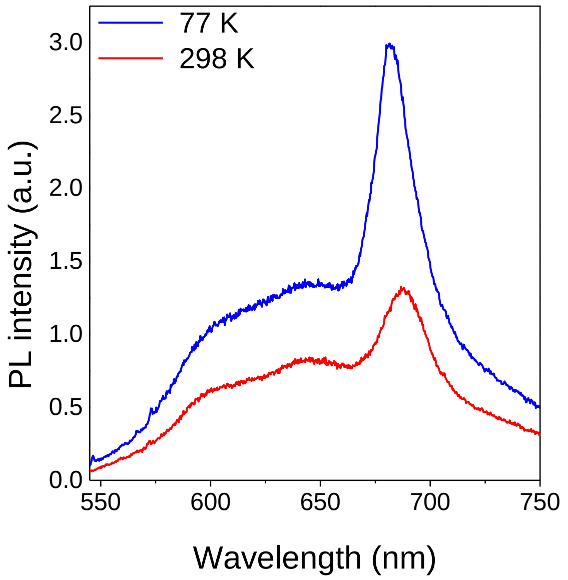

Figure S14 | Temperature dependent PL measurements from 2L PEN. PL spectra from 2L regime of PEN samples. The sharp PL emission at 680 nm is visible from 2L but coupled with high-energy CT emissions. The FWHM at 298 K from 2L is ~49 nm and at 77 K is ~30 nm, which is much broader as compared to 1L.

Table S1: Diffusion coefficients from various organic/in-organic materials: experimental values

Material Type	Material Name	Diffusion Coefficient (*cm^2 sec^{-1}*)	Temperature (*K*)
Inorganic systems	GaAs Quantum wells[42]	0.1-10	4.2
	1L MoS_2[43]	0.05-0.1	298
	1L WSe_2[44]	14.5	298
	1L WS_2[45]	0.41	298
	1L $MoTe_2$[46]	0.1	70
	Phosphorene [47]	5 E-3	4
	Carbon Nanotubes[48]	44	298
	WS_2-tetracene heterostructure[49]	1	298
	WSe_2-thin film/bulk[50]	15/9	298
Organic systems	C8S3-J aggregates[27]	70	298
	Light harvesting nanotubes (LHNs-amphiphilic cyanine dyes)[26]	55	298
	Meso-tetra(4-sulfonatophenyl) porphyrin[25]	3-6	298
	Tetracene thin film[51]	2.8	298
	Poly-3 hexylthiophene[52]	1.8 E-3	298
	C_{70} thin film[53]	3.5 E-3	77
	Pentacene thin films[54]	2.5 E-2	298
This work	2D Pentacene-WL	2.4	298
		3.5	77
	2D Pentacene-1L	346.9	298
		354.5	77

References

1 Pope, M. & Swenberg, C. E. Electronic processes in organic solids. *Annual Review of Physical Chemistry* **35**, 613-655 (1984).

2 Kasha, M. Energy Transfer Mechanisms and the Molecular Exciton Model for Molecular Aggregates. *Radiation Research* **20**, 55-70, doi:doi:10.2307/3571331 (1963).

3 Davydov, A. Theory of molecular excitations. *Plenum press, New York* **7**, 387-394 (1971).

4 Spano, F. C. & Silva, C. H-and J-aggregate behavior in polymeric semiconductors. *Annual review of physical chemistry* **65**, 477-500 (2014).

5 Grégoire, P. *et al.* Excitonic coupling dominates the homogeneous photoluminescence excitation linewidth in semicrystalline polymeric semiconductors. *arXiv preprint arXiv:1702.03378* (2017).

6 Braslavsky, S. E. Glossary of terms used in photochemistry, (IUPAC Recommendations 2006). *Pure and Applied Chemistry* **79**, 293-465 (2007).

7 Robinson, J. W. *Atomic spectroscopy*. (CRC Press, 1996).

8 Zhao, H., Moehl, S. & Kalt, H. Coherence length of excitons in a semiconductor quantum well. *Physical review letters* **89**, 097401 (2002).

9 Tanaka, S. *et al.* Enhancement of the Exciton Coherence Size in Organic Semiconductor by Alkyl Chain Substitution. *The Journal of Physical Chemistry C* **120**, 7941-7948 (2016).

10 Spano, F. C. & Yamagata, H. Vibronic Coupling in J-Aggregates and Beyond: A Direct Means of Determining the Exciton Coherence Length from the Photoluminescence Spectrum. *The Journal of Physical Chemistry B* **115**, 5133-5143, doi:10.1021/jp104752k (2011).

11 Stehr, V., Fink, R., Tafipolski, M., Deibel, C. & Engels, B. Comparison of different rate constant expressions for the prediction of charge and energy transport in oligoacenes. *Wiley Interdisciplinary Reviews: Computational Molecular Science* **6**, 694-720 (2016).

12 Spano, F. C. The Spectral Signatures of Frenkel Polarons in H- and J-Aggregates. *Accounts of Chemical Research* **43**, 429-439, doi:10.1021/ar900233v (2010).

13 Hestand, N. *et al.* Polarized Absorption in Crystalline Pentacene: Theory vs Experiment. *The Journal of Physical Chemistry C* **119**, 22137-22147 (2015).

14 Hoffmann, M. *et al.* The lowest energy Frenkel and charge-transfer excitons in quasi-one-dimensional structures: application to MePTCDI and PTCDA crystals. *Chemical Physics* **258**, 73-96 (2000).

15 Chua, L.-L. *et al.* General observation of n-type field-effect behaviour in organic semiconductors. *Nature* **434**, 194 (2005).

16 Lee, K. *et al.* Interfacial Trap Density-of-States in Pentacene-and ZnO-Based Thin-Film Transistors Measured via Novel Photo-excited Charge-Collection Spectroscopy. *Advanced Materials* **22**, 3260-3265 (2010).

17 Podzorov, V. *et al.* Intrinsic charge transport on the surface of organic semiconductors. *Physical review letters* **93**, 086602 (2004).

18 Bardeen, C. J. Excitonic processes in molecular crystalline materials. *MRS Bulletin* **38**, 65-71 (2013).

19 Rogers, J. A. Toward paperlike displays. *Science* **291**, 1502-1503 (2001).
20 Sirringhaus, H., Tessler, N. & Friend, R. H. Integrated optoelectronic devices based on conjugated polymers. *Science* **280**, 1741-1744 (1998).
21 Hoffmann, M. *et al.* The lowest energy Frenkel and charge-transfer excitons in quasi-one-dimensional structures: application to MePTCDI and PTCDA crystals. *Chemical Physics* **258**, 73-96 (2000).
22 Zhu, T., Wan, Y. & Huang, L. Direct Imaging of Frenkel Exciton Transport by Ultrafast Microscopy. *Accounts of chemical research* **50**, 1725-1733 (2017).
23 Mikhnenko, O. V., Blom, P. W. & Nguyen, T.-Q. Exciton diffusion in organic semiconductors. *Energy & Environmental Science* **8**, 1867-1888 (2015).
24 Jang, S., Newton, M. D. & Silbey, R. J. Multichromophoric Förster resonance energy transfer. *Physical Review Letters* **92**, 218301 (2004).
25 Wan, Y., Stradomska, A., Knoester, J. & Huang, L. Direct Imaging of Exciton Transport in Tubular Porphyrin Aggregates by Ultrafast Microscopy. *Journal of the American Chemical Society* **139**, 7287-7293 (2017).
26 Caram, J. R. *et al.* Room-Temperature Micron-Scale Exciton Migration in a Stabilized Emissive Molecular Aggregate. *Nano letters* **16**, 6808-6815 (2016).
27 Clark, K. A., Krueger, E. L. & Vanden Bout, D. A. Direct measurement of energy migration in supramolecular carbocyanine dye nanotubes. *The journal of physical chemistry letters* **5**, 2274-2282 (2014).
28 Dicke, R. H. Coherence in spontaneous radiation processes. *Physical Review* **93**, 99 (1954).
29 Cong, K. *et al.* Dicke superradiance in solids. *JOSA B* **33**, C80-C101 (2016).
30 Gross, M. & Haroche, S. Superradiance: An essay on the theory of collective spontaneous emission. *Physics reports* **93**, 301-396 (1982).
31 Bradac, C. *et al.* Room-temperature spontaneous superradiance from single diamond nanocrystals. *Nature communications* **8**, 1205 (2017).
32 Scheibner, M. *et al.* Superradiance of quantum dots. *Nature Physics* **3**, 106 (2007).
33 Abasto, D. F., Mohseni, M., Lloyd, S. & Zanardi, P. Exciton diffusion length in complex quantum systems: the effects of disorder and environmental fluctuations on symmetry-enhanced supertransfer. *Phil. Trans. R. Soc. A* **370**, 3750-3770 (2012).
34 Sim, M. *et al.* Dependence of Exciton Diffusion Length on Crystalline Order in Conjugated Polymers. *The Journal of Physical Chemistry C* **118**, 760-766, doi:10.1021/jp409776s (2014).
35 Lunt, R. R., Benziger, J. B. & Forrest, S. R. Relationship between crystalline order and exciton diffusion length in molecular organic semiconductors. *Advanced Materials* **22**, 1233-1236 (2010).
36 Lunt, R. R., Giebink, N. C., Belak, A. A., Benziger, J. B. & Forrest, S. R. Exciton diffusion lengths of organic semiconductor thin films measured by spectrally resolved photoluminescence quenching. *Journal of Applied Physics* **105**, 053711 (2009).
37 Zhang, Y. *et al.* Probing Carrier Transport and Structure-Property Relationship of Highly Ordered Organic Semiconductors at the Two-Dimensional Limit. *Physical Review Letters* **116**, 016602 (2016).
38 Haedler, A. T. *et al.* Long-range energy transport in single supramolecular nanofibres

at room temperature. *Nature* **523**, 196 (2015).
39 Wu, S., Cheng, L. & Wang, Q. Excitonic effects and related properties in semiconductor nanostructures: roles of size and dimensionality. *Materials Research Express* **4**, 085017 (2017).
40 Hong, X. *et al.* Ultrafast charge transfer in atomically thin MoS2/WS2 heterostructures. *Nat Nano* **9**, 682-686, doi:10.1038/nnano.2014.167 (2014).
41 Jariwala, D. *et al.* Hybrid, Gate-Tunable, van der Waals p–n Heterojunctions from Pentacene and MoS2. *Nano Letters* **16**, 497-503, doi:10.1021/acs.nanolett.5b04141 (2016).
42 Vörös, Z., Balili, R., Snoke, D., Pfeiffer, L. & West, K. Long-distance diffusion of excitons in double quantum well structures. *Physical review letters* **94**, 226401 (2005).
43 Sun, D. *et al.* Observation of rapid exciton–exciton annihilation in monolayer molybdenum disulfide. *Nano letters* **14**, 5625-5629 (2014).
44 Cadiz, F. *et al.* Exciton diffusion in WSe2 monolayers embedded in a van der Waals heterostructure. *Applied Physics Letters* **112**, 152106 (2018).
45 Yuan, L. & Huang, L. Exciton dynamics and annihilation in WS 2 2D semiconductors. *Nanoscale* **7**, 7402-7408 (2015).
46 Froehlicher, G., Lorchat, E. & Berciaud, S. Direct versus indirect band gap emission and exciton-exciton annihilation in atomically thin molybdenum ditelluride (MoTe 2). *Physical Review B* **94**, 085429 (2016).
47 Surrente, A. *et al.* Onset of exciton-exciton annihilation in single-layer black phosphorus. *Physical Review B* **94**, 075425 (2016).
48 Moritsubo, S. *et al.* Exciton diffusion in air-suspended single-walled carbon nanotubes. *Physical review letters* **104**, 247402 (2010).
49 Zhu, T. *et al.* Highly mobile charge-transfer excitons in two-dimensional WS2/tetracene heterostructures. *Science Advances* **4**, eaao3104 (2018).
50 Cui, Q., Ceballos, F., Kumar, N. & Zhao, H. Transient absorption microscopy of monolayer and bulk WSe2. *ACS nano* **8**, 2970-2976 (2014).
51 Wan, Y. *et al.* Cooperative singlet and triplet exciton transport in tetracene crystals visualized by ultrafast microscopy. *Nature chemistry* **7**, 785 (2015).
52 Shaw, P. E., Ruseckas, A. & Samuel, I. D. Exciton diffusion measurements in poly (3-hexylthiophene). *Advanced Materials* **20**, 3516-3520 (2008).
53 Kozlov, O. V. *et al.* Real-time tracking of singlet exciton diffusion in organic semiconductors. *Physical review letters* **116**, 057402 (2016).
54 Marciniak, H., Pugliesi, I., Nickel, B. & Lochbrunner, S. Ultrafast singlet and triplet dynamics in microcrystalline pentacene films. *Physical Review B* **79**, 235318 (2009).
